# ﻿Two new species and two new records of *Homidia* (Collembola, Entomobryidae) from China

**DOI:** 10.3897/zookeys.1135.89373

**Published:** 2022-12-15

**Authors:** Mei-dong Jing, Yi-tong Ma

**Affiliations:** 1 School of Life Sciences, Nantong University, Nantong, Jiangsu 226000, China Nantong University Nantong China

**Keywords:** Chaetotaxy, Entomobryinae, Jiangxi, taxonomy

## Abstract

*Homidia*, one of the largest genera of the family Entomobryidae, is widely distributed in China. To date, 46 species of this genus are present in China and account for approximately 60 % of all known species of the genus. In the present paper, two new species of *Homidia* are described from China: *H.acutus***sp. nov.** and *H.changensis***sp. nov.** The former is discriminated by the brown to blue-violet pigment present on whole dorsal body and by pointed tenent hairs. The latter is characterised by having only scattered traces of brown pigment on tergites, and by the special macrochaetal formula of coxae. Additionally, two known species of the genus, *H.linhaiensis* Shi, Pan & Qi, 2009 and *H.socia* Denis, 1929, are reported from Jiangxi Province for the first time, and some of their taxonomic characters are described. A key to the Chinese species of the genus is provided.

## ﻿Introduction

*Homidia* was established as a subgenus of *Entomobrya* by Börner (1906) based on the presence of inner spines at the base of the dens in adults. Denis (1929) considered the character significant enough to raise *Homidia* to generic level. The genus is also characterised by the presence of “eyebrow” macrochaetae on the anterior part of Abd. IV in adults, the absence of scales, and a bidentate mucro with the subapical tooth much larger than the apical one.

Colour pattern plays a key role in classification of *Homidia* because intraspecific variability is very low. However, some species, such as *H.sauteri*, *H.similis*, *H.sinensis*, and *H.socia*, are widespread and some intraspecific variability of colour pattern from different regions may be sometimes present. Chaetotaxy is also very useful in species identification, especially that of Abd. I, IV, and labium basis.

**Figure 1.**
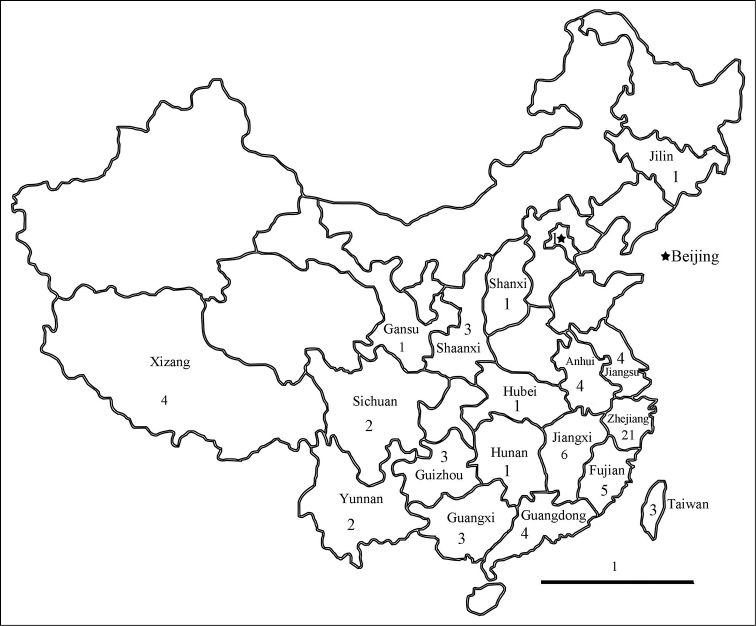
Distribution of all Chinese species of *Homidia* (the number in each region represents the number of the species reported from this province). Scale bar: 1000 km.

The first person to study the Chinese *Homidia* was the Frenchman J. R. Denis, who reported *Homidiasauteri* (Börner, 1909) from Yunnan Province in 1928. To date, 46 species have been described or reported from China among a total of 75 species worldwide (Bellinger et al. 1996–2022, Table 1). These species are mainly distributed in the eastern region of China, especially Zhejiang Province (Fig. 1).

**Table 1.** Species checklist of *Homidia* recorded from China.

Species name	Distribution
*H.acutus* sp. nov.	Jiangxi*
*H.anhuiensis* Li & Chen, 1997	Anhui
*H.apigmenta* Shi, Pan & Zhang, 2010	Fujian
*H.breviseta* Pan, 2022	Xizang
*H.changensis* sp. nov.	Jiangxi*
*H.chroma* Pan & Yang, 2019	Guangdong
*H.dianbaiensis* (Lin, 1985)	Guangdong
*H.emeiensis* Jia, Chen & Christiansen, 2004	Sichuan
*H.fascia* Wang & Chen, 2001	Jiangsu
*H.formosana* Uchida, 1943	Taiwan; Zhejiang
*H.hangzhouensis* Pan & Ma, 2021	Zhejiang
*H.hexaseta* Pan, Shi & Zhang, 2011	Zhejiang
*H.huashanensis* Jia, Chen & Christiansen, 2005	Shaanxi
*H.jordanai* Pan, Shi & Zhang, 2011	Zhejiang
*H.laha* Christiansen & Bellinger, 1992	Zhejiang
*H.latifolia* Chen & Li, 1999	Zhejiang
*H.leei* Chen & Li, 1997	Jiangxi
*H.leniseta* Pan & Yang, 2019	Guangdong
*H.linhaiensis* Shi, Pan & Qi, 2009	Jiangxi*; Zhejiang
*H.maijiensis* Zhou & Ma, 2022	Gansu
*H.mediofascia* Shi, Pan & Bai, 2009	Shaanxi
*H.nigrifascia* Ma & Pan, 2017	Guizhou
*H.nigrocephala* Uchida, 1943	Taiwan
*H.obliquistria* Ma & Pan, 2017	Guizhou
*H.pentachaeta* Li & Christiansen, 1997	Jiangsu
*H.phjongjangica* Szeptycki, 1973	Jilin; Zhejiang
*H.polyseta* Chen, 1998	Hunan
*H.pseudofascia* Pan, Zhang & Li, 2015	Jiangsu
*H.pseudosinensis* Shi & Pan, 2012	Fujian
*H.qimenensis* Yi & Chen, 1999	Anhui; Fujian; Guangxi; Jiangxi; Zhejiang
*H.quadriseta* Pan, 2018	Zhejiang
*H.quadrimaculata* Pan, 2015	Zhejiang
*H.sauteri* (Börner, 1909)	Shanxi; Yunnan; Zhejiang
*H.sichuanensis* Jia, Zhang & Jordana, 2010	Sichuan; Guangdong; Guangxi; Guizhou; Xizang
*H.similis* Szeptycki, 1973	Zhejiang
*H.sinensis* Denis, 1929	Beijing; Fujian; Yunnan; Zhejiang; Xizang
*H.socia* Denis, 1929	Anhui; Fujian; Guangxi; Jiangsu; Jiangxi*; Taiwan; Zhejiang
*H.taibaiensis* Yuan & Pan, 2013	Shaanxi
*H.tiantaiensis* Chen & Lin, 1998	Zhejiang
*H.tibetensis* Chen & Zhong, 1998	Xizang
*H.transitoria* Denis, 1929	Fujian
*H.triangulimacula* Pan & Shi, 2015	Zhejiang
*H.unichaeta* Pan, Shi & Zhang, 2010	Zhejiang
*H.wanensis* Pan & Ma, 2021	Anhui
*H.xianjuensis* Wu & Pan, 2016	Zhejiang
*H.yandangensis* Pan, 2015	Zhejiang
*H.zhangi* Pan & Shi, 2012	Zhejiang
*H.ziguiensis* Jia, Chen & Christiansen, 2003	Hubei

Notes:* described or reported in this paper.

## ﻿Materials and methods

Specimens were collected with an aspirator and stored in 99 % alcohol. They were mounted on glass slides in Marc André II solution, and were studied with a Leica DM2500 phase contrast microscope. Photographs were taken with a Leica DFC300 FX digital camera mounted on the microscope and a ZEISS Gemini SEM 300. They were enhanced with Photoshop CS2 (Adobe Inc.). The nomenclature of the dorsal macrochaetotaxy of head and interocular chaetae are described following Szeptycki (1973) and Mari Mutt (1986). Labial chaetae are designated following Gisin (1964) and tergal chaetae of the body after Szeptycki (1979).

### ﻿Abbreviations

**Abd** abdominal segment;

**Ant** antennal segment;

**asl** above sea level;

**mac** macrochaeta(e);

**ms** specialised microchaeta(e);

**NTU** Nantong University;

**sens** specialised ordinary chaeta(e);

**Th** thoracic segment.

## ﻿Taxonomic account

### ﻿ Homidia acutus sp. nov.

Taxon classificationAnimaliaCollembolaEntomobryidae

856FBA28-CBAA-598F-ACEE-7B3ED53B3296

https://zoobank.org/C2B263A9-3D3D-4BEE-A1AF-2B04559281E6

Figs 2–5 , 6–13 , 14–20 , 21–29 , 30, 31 , 32 , 33 , 34–40 , Table 2

#### Type material.

***Holotype*.** 1♀ on slide, **China**, Jiangxi Province, Pingxiang City, Luxi County, Gate of Wugong Mountain, 27°29'27"N, 114°07'33"E, 393 m asl, sample number 1229, collected by Y-T Ma, 7-XI-2020, deposited in NTU. ***Paratypes*.** 3♀ on slides, same data as holotype.

#### Descriptions.

***Size*.** Body length up to 2.05 mm.

**Figures 2–5.**
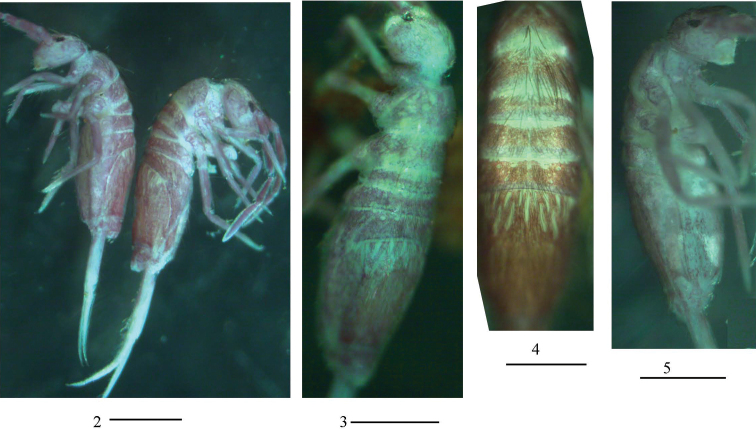
Habitus of *Homidiaacutus* sp. nov. **2** lateral view **3, 4** dorsal view **5** ventral view. Scale bars: 500 μm.

***Colouration*.** Ground colour pale white to pale yellow. Eye patches dark blue. Brown to blue-violet pigment present on whole dorsal body, antennae, legs, ventral tube, and manubrium. Some unpigmented irregular stripes or spots present on dorsal side of body (Figs 2–5).

**Figures 6–13.**
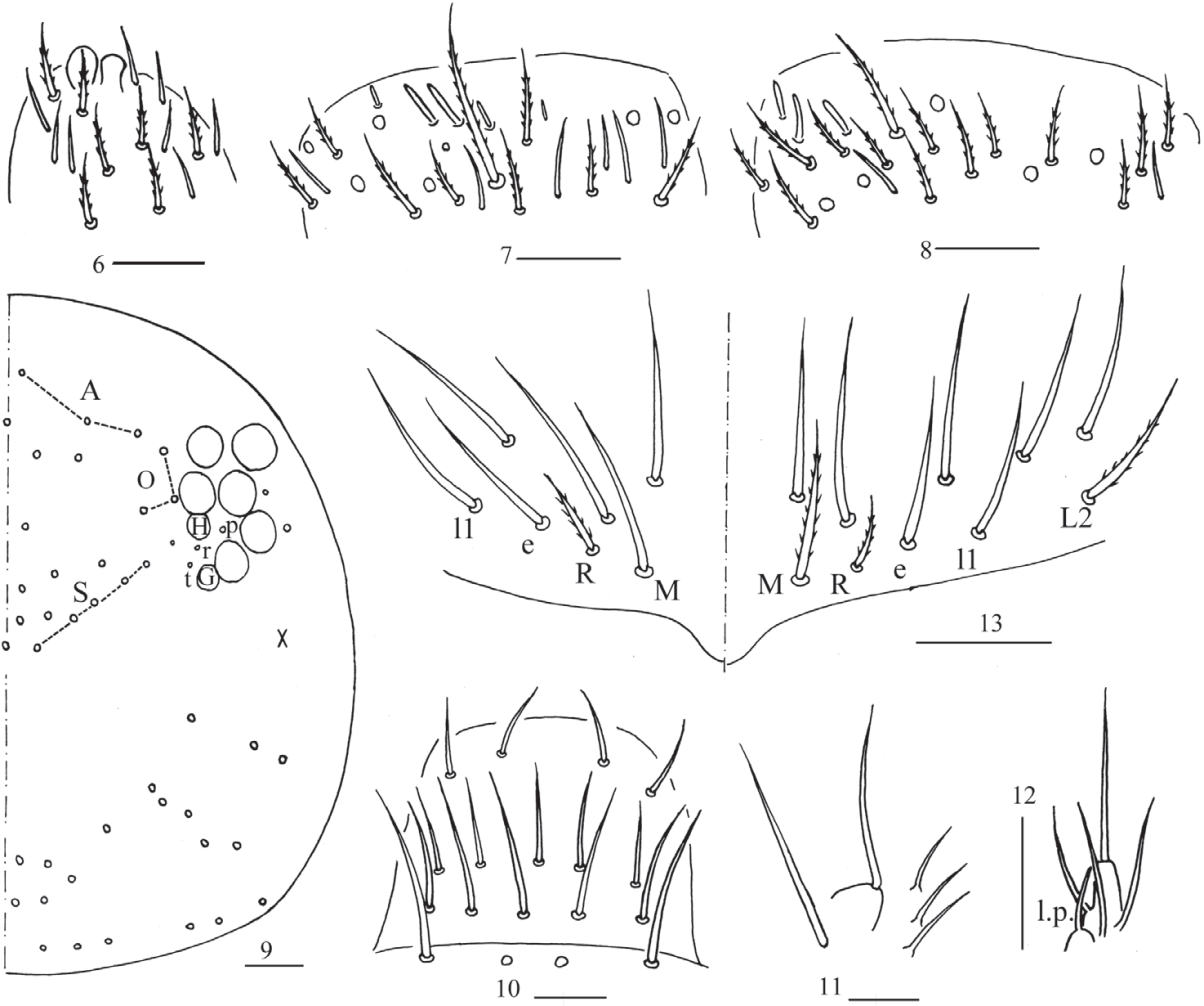
*Homidiaacutus* sp. nov. **6** apex of Ant. IV **7**Ant. III organ **8** distal Ant. II **9** dorsal chaetotaxy of head **10** labrum **11** maxillary palp and outer lobe **12** labial palp E **13** labial base. Scale bars: 20 μm.

***Head*.** Antenna 0.46–0.58× body length; antennal segment ratio I: II: III: IV = 1: 1.35–1.67: 1.20–1.33: 1.88–1.93. Apical bulb of Ant. IV bilobed (Fig. 6). Ant. III organ with two rod-like chaetae (Fig. 7). Ant. II with three distal rod-like chaetae (Fig. 8). Eyes 8 + 8, G and H smaller than others; interocular chaetae with p, r, and t. Dorsal cephalic chaetotaxy with three antennal (A), three ocellar (O) and five sutural (S) mac (Fig. 9). Labral chaetae as 4/5, 5, 4, all smooth; labral papillae absent (Fig. 10). Basal chaeta of maxillary outer lobe thin, subequal to apical one; sublobal plate with three smooth chaeta-like processes (Fig. 11). Lateral process (l.p.) of labial papilla E differentiated, as thick as normal chaeta, with tip almost reaching apex of papilla E (Fig. 12). Chaetal formula of labial base as MRel_1_L_2_, chaetae e and l_1_ smooth, others ciliate, M of one side smooth in one individual, R/M as 0.45–0.60 (Fig. 13).

***Thorax*.**Th. II with four medio-medial (m1, m2, m2i, m2i2), three medio-sublateral (m4, m4i, m4p), 35–38 posterior mac, one ms and two sens (ms antero-internal to sens). Th. III with 40–49 mac and two sens (Fig. 14). Pseudopores on coxae not clearly seen; coxal macrochaetal formula as 4/4+1, 3/4+2 (Figs 15–17). Trochanteral organ with 40 smooth chaetae (Fig. 18). All tenent hairs pointed and shorter than inner edge of unguis. Unguis with three inner teeth, basal pair located at 0.39–0.42 distance from base of inner edge of unguis, distal unpaired tooth at 0.64–0.70 distance from base; unguiculus lanceolate, outer edge slightly serrate (Figs 19–31).

**Figures 14–20.**
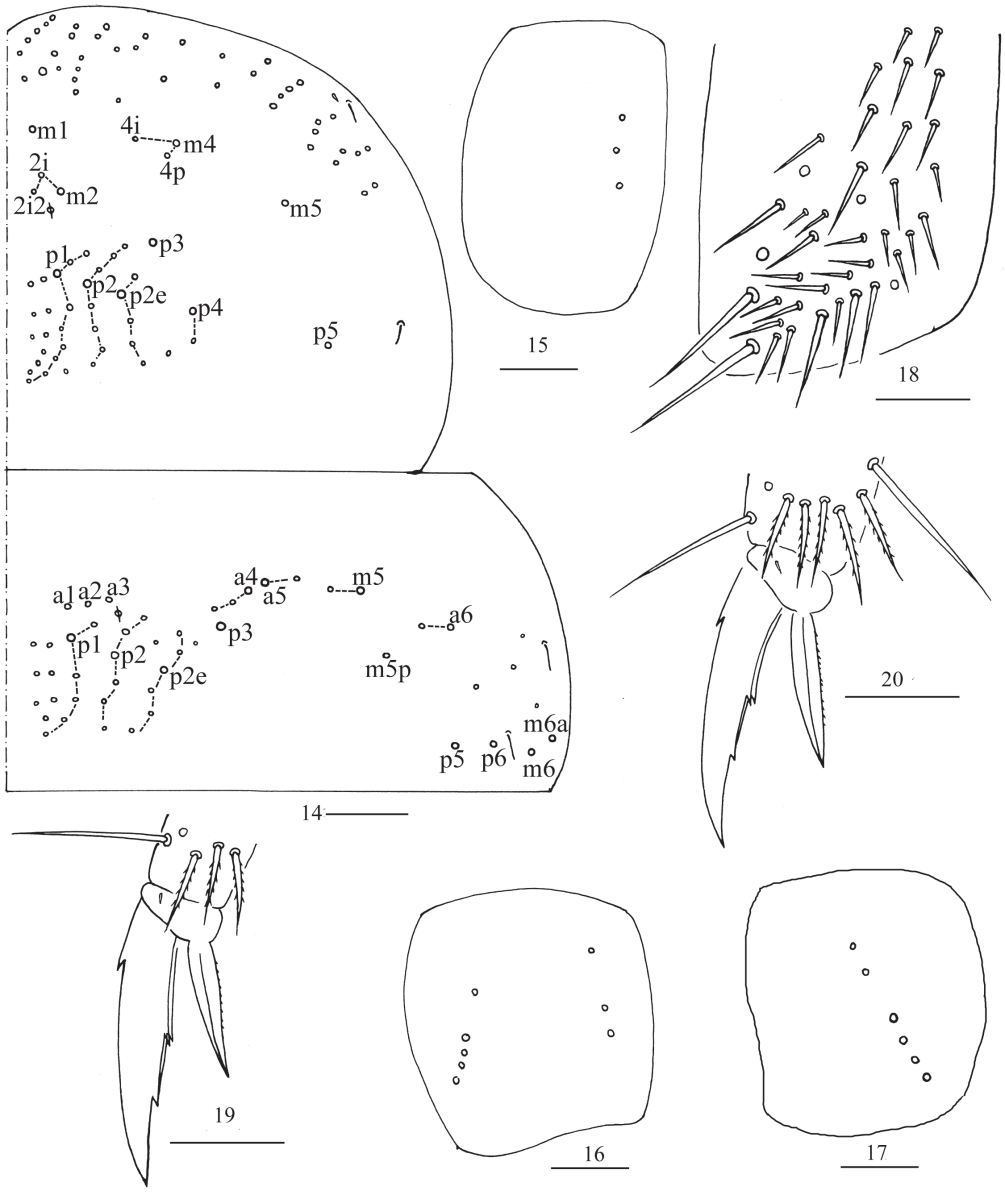
*Homidiaacutus* sp. nov. **14** cchaetotaxy of Th. II–III **15–17** coxal chaetotaxy of fore, middle and hind leg **18** trochanteral organ **19, 20** fore and hind foot complex. Scale bar: 50 μm (**14**); 20 μm (**15–20**).

**Figures 21–29.**
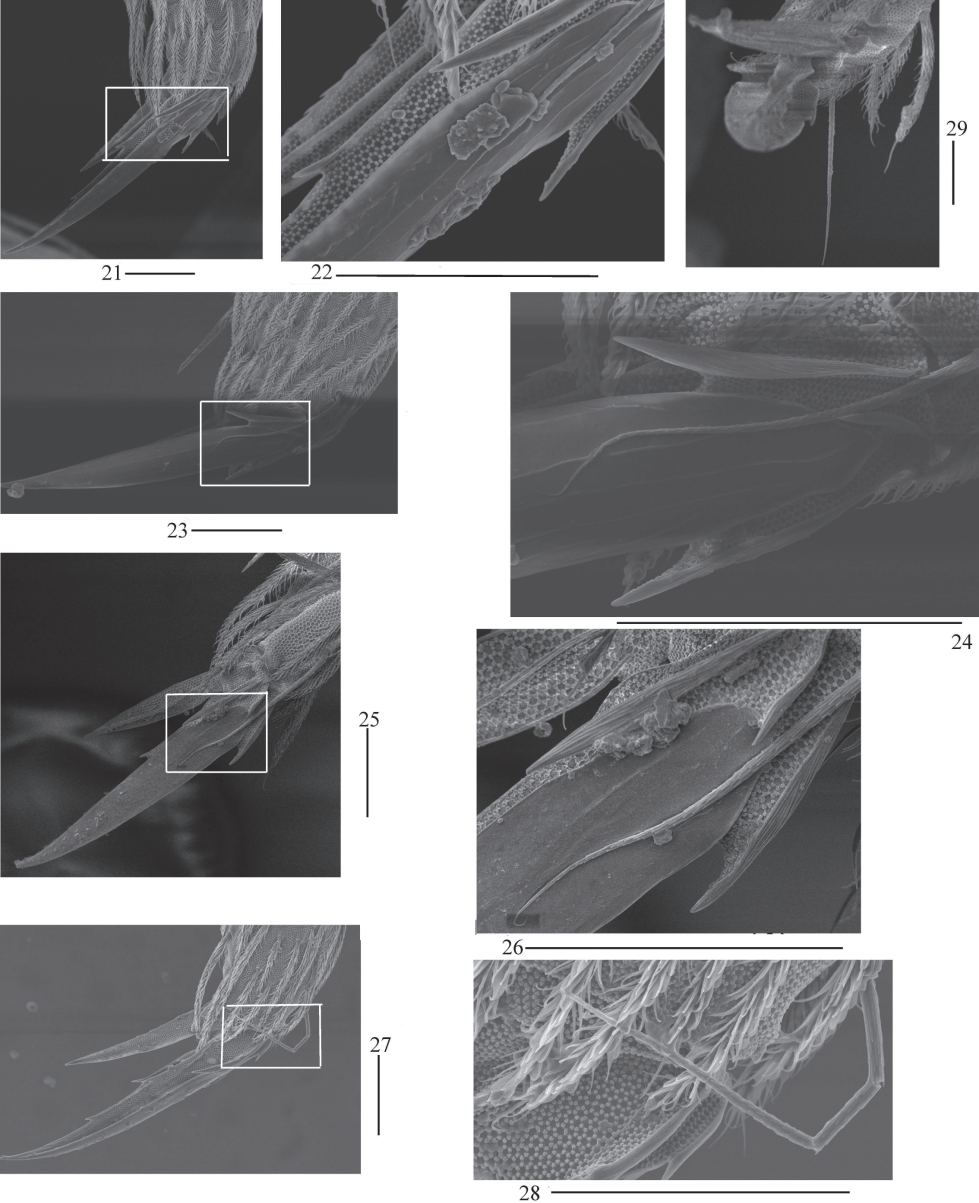
SEM photomicrographs of *Homidiaacutus* sp. nov. **21, 23, 25** fore foot complex of three individuals **22, 24, 26** magnifications of white rectangles of **21, 23, 25** respectively **27, 29** middle foot complex of two individuals **28** magnification of white rectangle of **27**. Scale bars: 20 μm.

**Figures 30, 31.**
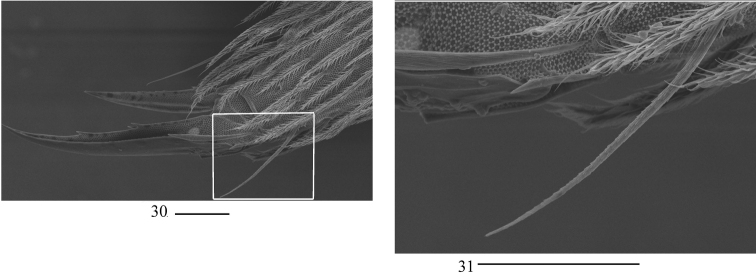
SEM photomicrographs of *Homidiaacutus* sp. nov. **30** hind foot complex **31** magnification of white rectangle of **30**. Scale bars: 20 μm.

**Figure 32.**
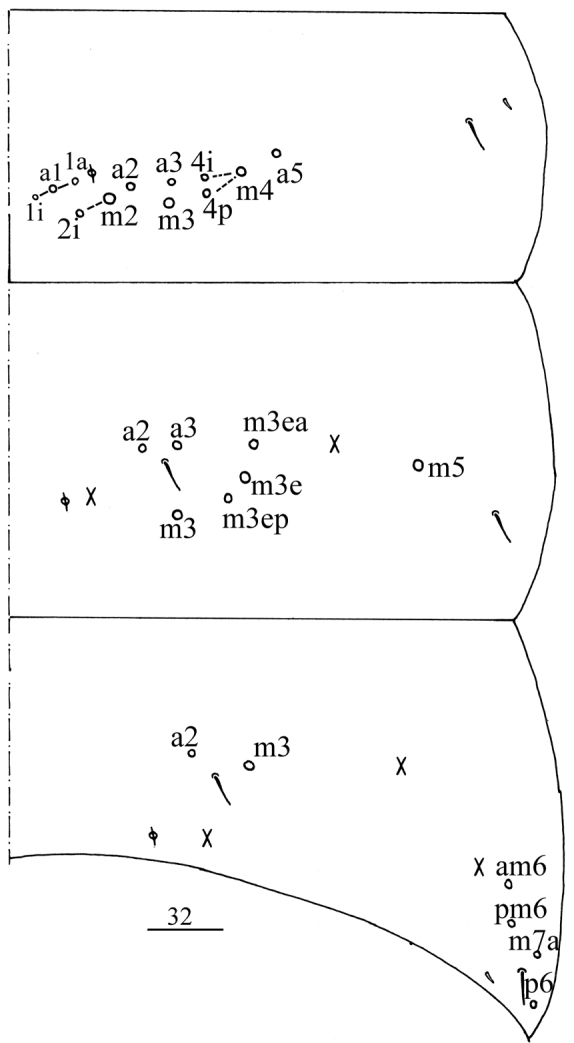
Chaetotaxy of Abd. I–III of *Homidiaacutus* sp. nov. Scale bar: 50 μm.

**Figure 33.**
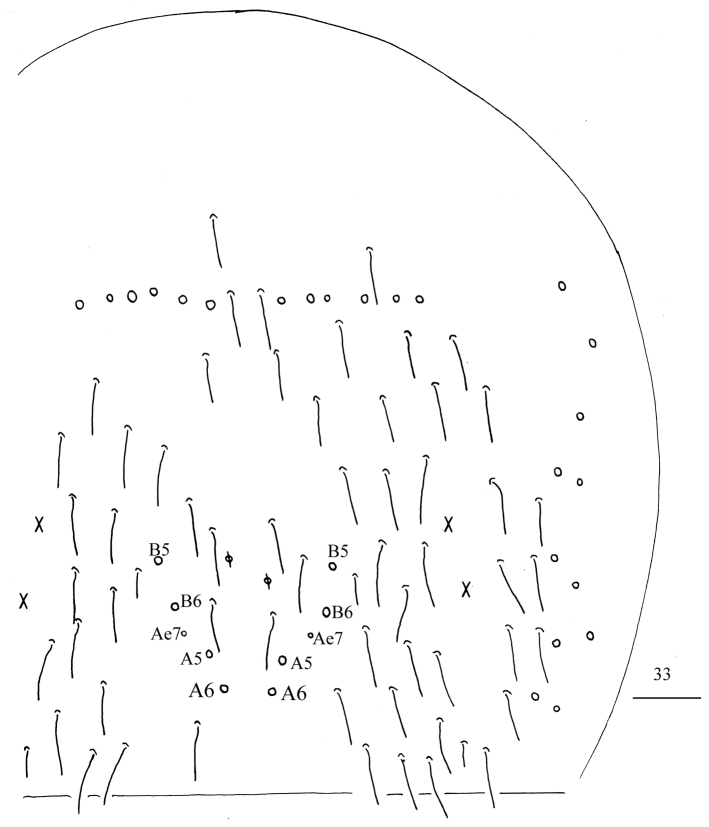
Chaetotaxy of Abd. IV of *Homidiaacutus* sp. nov. Scale bar: 50 μm.

**Figures 34–40.**
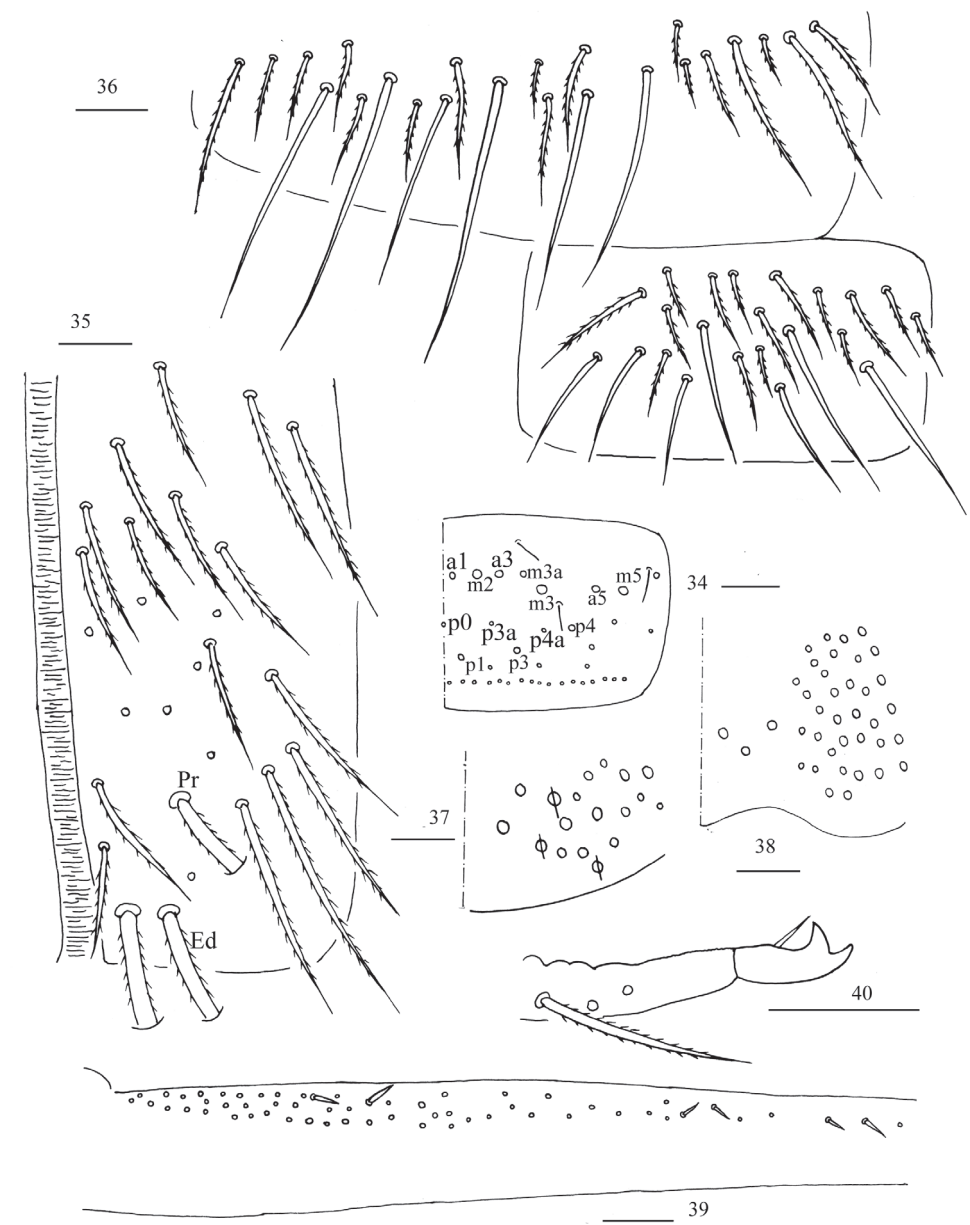
*Homidiaacutus* sp. nov. **34** chaetotaxy of Abd. V **35** anterior face of ventral tube **36** posterior face and lateral flap of ventral tube **37** manubrial plaque **38** ventro-apical part of manubrium **39** proximal section of dens (circles also representing spines) **40** mucro. Scale bars: 20 μm.

***Abdomen*.** Range of Abd. IV length as 5.00–7.50× as dorsal axial length of Abd. III. Abd. I with 11 or 12 (a1a, a1–3, a5, m2–4, m2i, m4i, m4p, a1i sometimes present) mac, ms antero-external to sens. Abd. II with six (a2, a3, m3, m3e, m3ea, m3ep) central, one (m5) lateral mac and two sens. Abd. III with two (a2, m3) central and four (am6, pm6, m7a, p6) lateral mac, one ms and two sens (Fig. 32). Abd. IV with two normal sens and approximately half length of elongate sens; anteriorly with six mac arranged in irregular transverse row, posteriorly with five central mac (A5, A6, B5, B6, Ae7), laterally with 12 or 13 mac (Fig. 33). Abd. V with three sens, middle one posterior to m3 (Fig. 34). Anterior face of ventral tube with 27–32 ciliate chaetae, 3+3 of them as mac, line connecting proximal (Pr) and external-distal (Ed) mac oblique to median furrow (Fig. 35); posterior face with six distal smooth and numerous ciliate chaetae; lateral flap with seven smooth and 10–15 ciliate chaetae (Fig. 36). Manubrial plate dorsally with 13–15 ciliate chaetae and three pseudopores (Fig. 37); ventrally with 32–38 ciliate chaetae on each side (Fig. 38). Dens with 32–59 smooth inner spines (Fig. 39). Mucro bidentate with subapical tooth larger than apical one; tip of basal spine reaching apex of subapical tooth; distal smooth section of dens almost equal to mucro in length (Fig. 40).

#### Ecology.

In the leaves litter of *Phyllostachysedulis*.

The name of the species is derived from the Latin *acutus* = pointed, which refers to the tip of tenent hairs.

#### Remarks.

The new species is characterised by pointed tip of tenent hairs and this character can be used to distinguish it from all known species of *Homidia*. It is similar to *H.zhangi* Pan & Shi, 2012 in colour pattern and labium, but there are some differences between them, such as tenent hairs, posterior chaetotaxy of Abd. IV, and other characters. The detailed character comparisons are listed in Table 2.

**Table 2.** Comparison between *H.acutus* sp. nov. and *H.zhangi*.

Characters	*H.acutus* sp. nov.	*H.zhangi*
Tip of tenent hairs	pointed	clavate
Mac m5 on Th. II	present	absent
Centro-posterior mac on Abd. IV	5 (A5, A6, B5, B6, Ae7)	3(4) (A6, B6, Ae7. B5 sometimes absent)
Inner teeth on unguis	3	4
Relative position of ms to sens on Abd. I	antero-external	antero-internal
Relative position of middle sens to m3 on Abd. V	postero-external	antero-external

### ﻿ Homidia changensis sp. nov.

Taxon classificationAnimaliaCollembolaEntomobryidae

D5E5314F-82F7-5C92-9CD5-1BF0F0FA7359

https://zoobank.org/7A88BCD3-07D5-40B4-8E44-BE1F21AD6FC8

Figs 41–42 , 43–49 , 50–55 , 56 , 57 , 58–64 , Table 3

#### Type material.

***Holotype*.** 1♀ on slide, **China**, Jiangxi Province, Nanchang City, Xinjian District, Jiuxi, 28°47'56"N, 115°45'11"E, 168 m asl, sample number 1243, collected by Y-T Ma, 12-XI-2020, deposited in NTU. ***Paratypes*.** 2♀ on slides, same data as holotype.

#### Description.

***Size*.** Body length up to 2.33 mm.

**Figures 41, 42.**
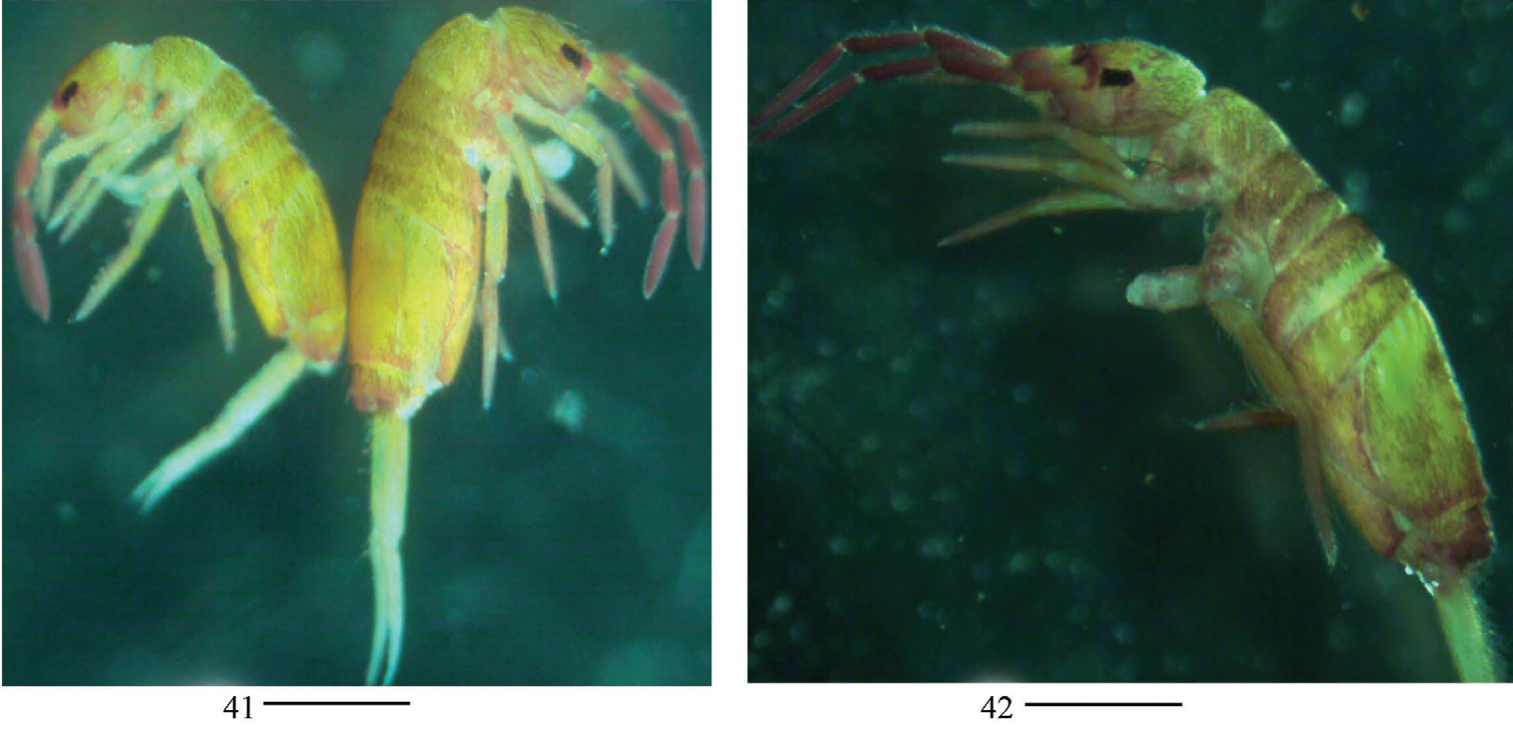
Habitus of *Homidiachangensis* sp. nov. Scale bars: 500 μm.

***Colouration*.** Ground colour yellow. Ant. II–IV and distal part of Ant. I brown. Eye patches dark blue. Coxae, tibiotarsi, posterior part of Abd. IV and Abd. V with scattered brown pigment (Figs 41, 42).

**Figures 43–49.**
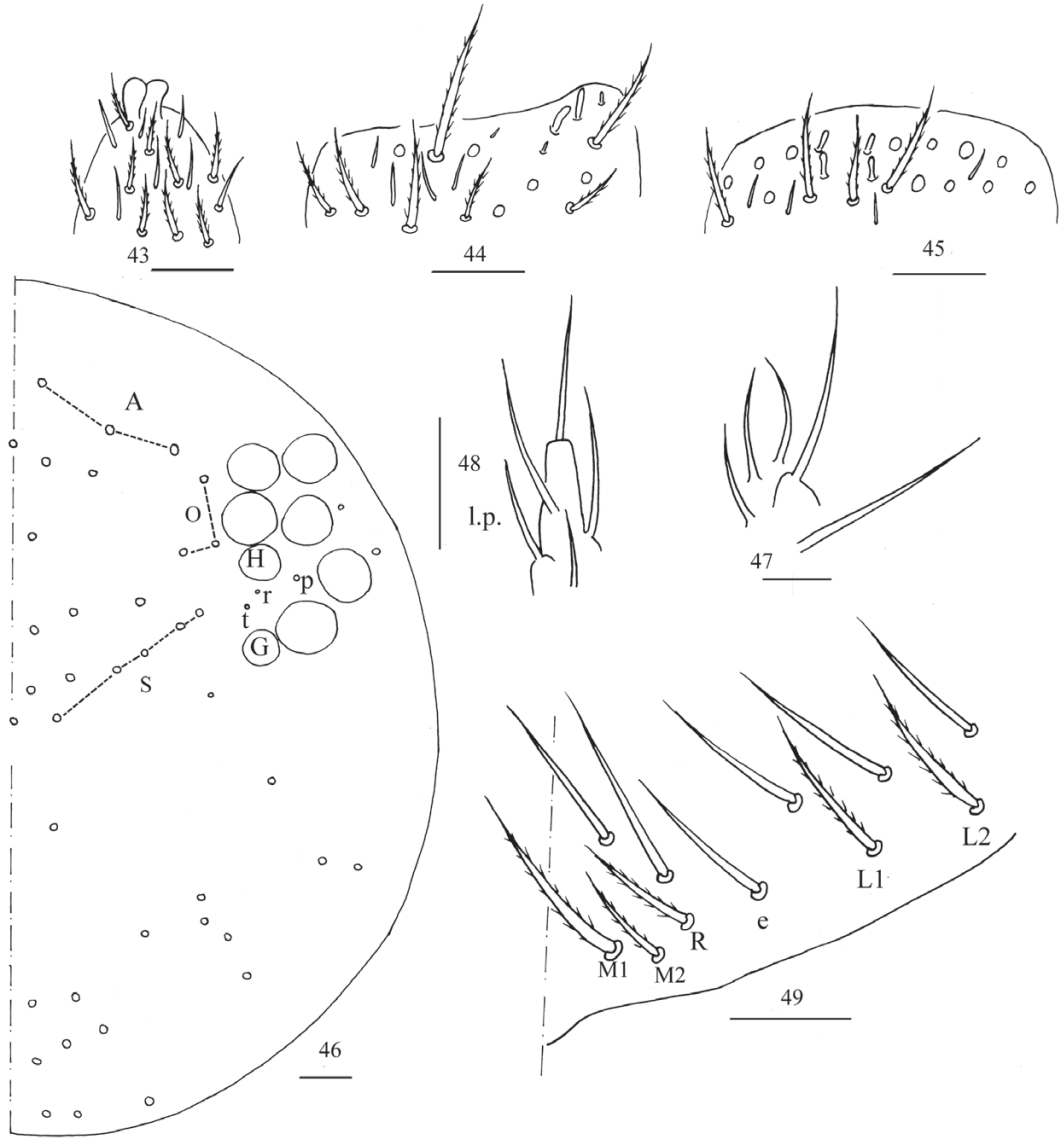
*Homidiachangensis* sp. nov. **43** apex of Ant. IV **44**Ant. III organ **45** distal Ant. II **46** dorsal chaetotaxy of head **47** maxillary palp and outer lobe **48** labial palp E **49** labial base. Scale bars: 20 μm.

***Head*.** Antenna 0.50–0.57× body length; antennal segment ratio I: II: III: IV = 1: 1.33–1.50: 1.17–1.30: 1.90–2.00. Apical bulb of Ant. IV bilobed (Fig. 43). Ant. III organ with two rod-like chaetae (Fig. 44). Ant. II with four distal rod-like chaetae (Fig. 45). Eyes 8 + 8, G and H smaller than others; interocular chaetae with p, r, and t. Dorsal cephalic chaetotaxy with three antennal (A), three ocellar (O) and five sutural (S) mac (Fig. 46). Basal chaeta of maxillary outer lobe thin, subequal to apical one; sublobal plate with three smooth chaeta-like processes (Fig. 47). Lateral process (l.p.) of labial papilla E differentiated, as thick as normal chaeta, with tip almost reaching apex of papilla E (Fig. 48). Chaetal formula of labial base as M_1_M_2_ReL_1_L_2_, chaeta e smooth, others ciliate, R/M_1_ as 0.63–0.70 (Fig. 49).

**Figures 50–55.**
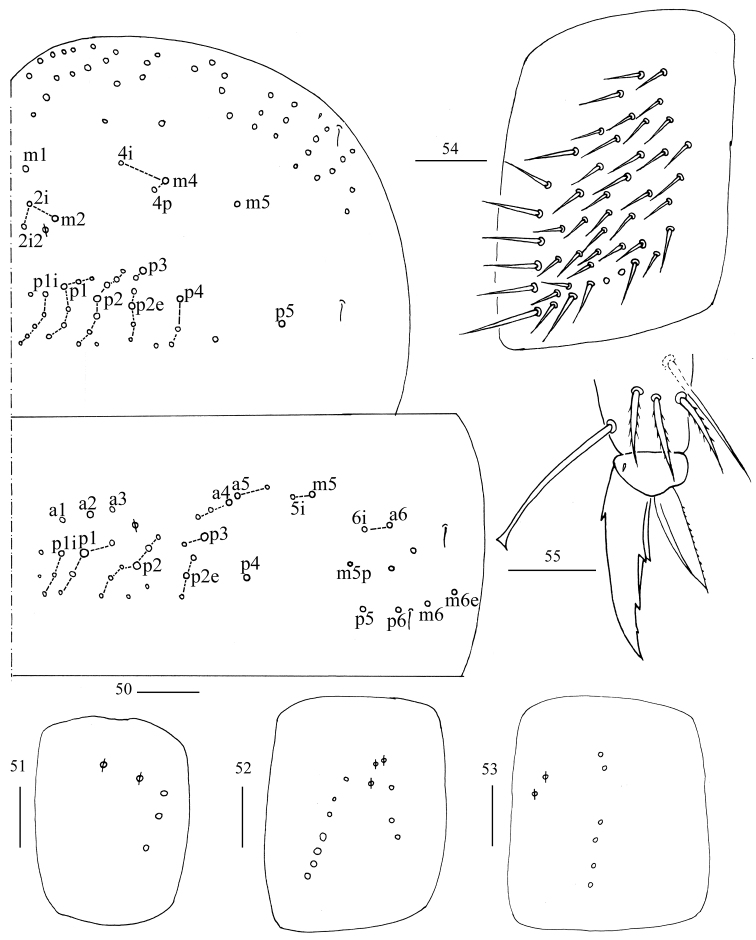
*Homidiachangensis* sp. nov. **50** chaetotaxy of Th. II–III **51–53** coxal chaetotaxy of fore, middle and hind leg **54** trochanteral organ **55** hind foot complex. Scale bar: 50 μm (**50**); 20 μm (**51–55**).

***Thorax***. Th. II with four medio-medial (m1, m2, m2i, m2i2), three medio-sublateral (m4, m4i, m4p), 32–38 posterior mac, one ms and two sens (ms antero-internal to sens). Th. III with 38–47 mac and two sens (Fig. 50). Pseudopores on coxa I–III as 2, 3, 2, respectively; coxal macrochaetal formula as 3/4+3(4), 3/4+2 (Figs 51–53). Trochanteral organ with 45–48 smooth chaetae (Fig. 54). Tenent hairs clavate and almost equal to inner edge of unguis. Unguis with four inner teeth, basal pair located at 0.31–0.41 distance from base of inner edge of unguis, distal unpaired teeth at 0.63–0.71 and 0.83–0.84 distance from base; unguiculus lanceolate, outer edge slightly serrate (Fig. 55).

**Figure 56.**
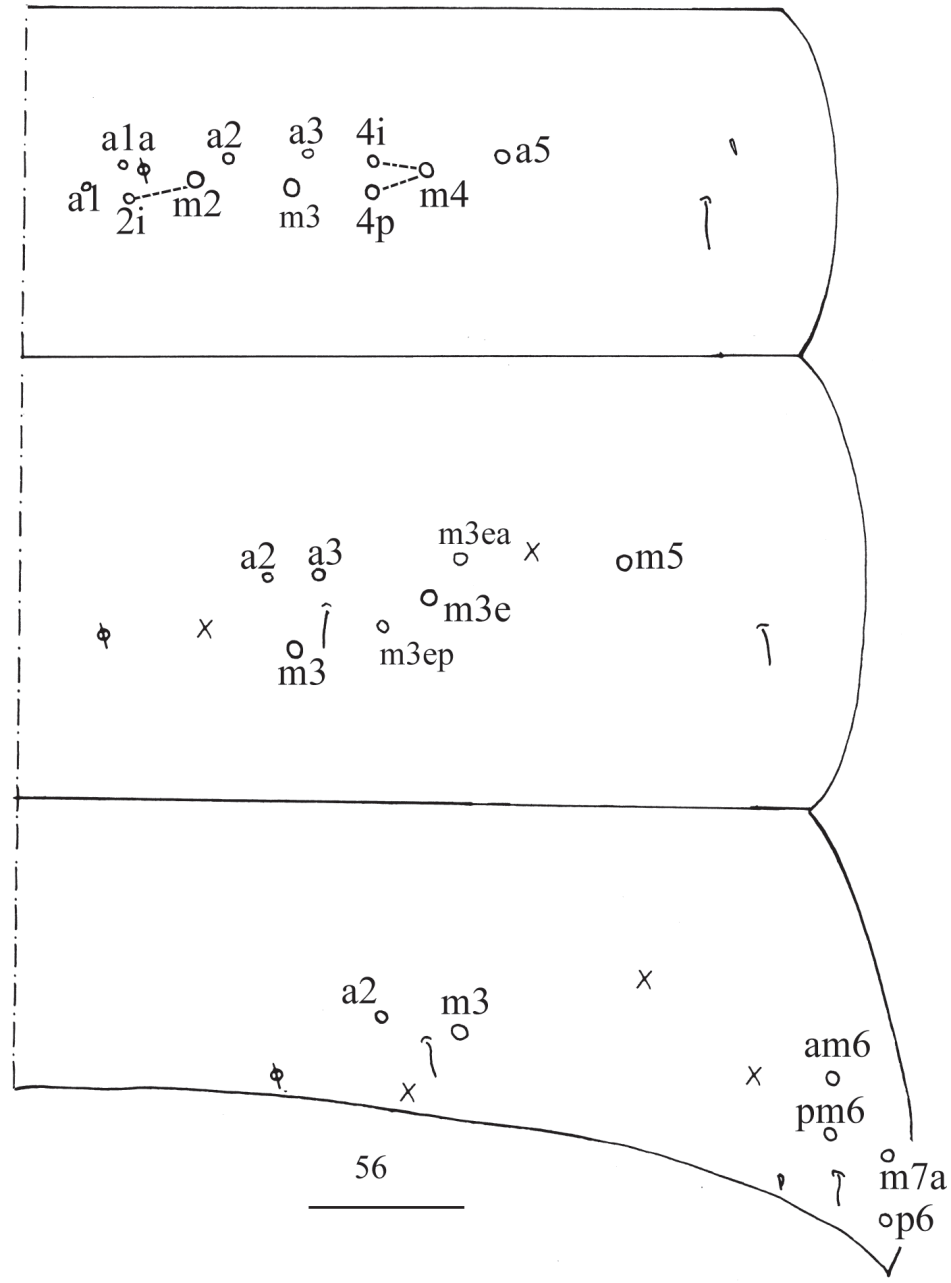
Chaetotaxy of Abd. I–III of *Homidiachangensis* sp. nov. Scale bar: 50 μm.

**Figure 57.**
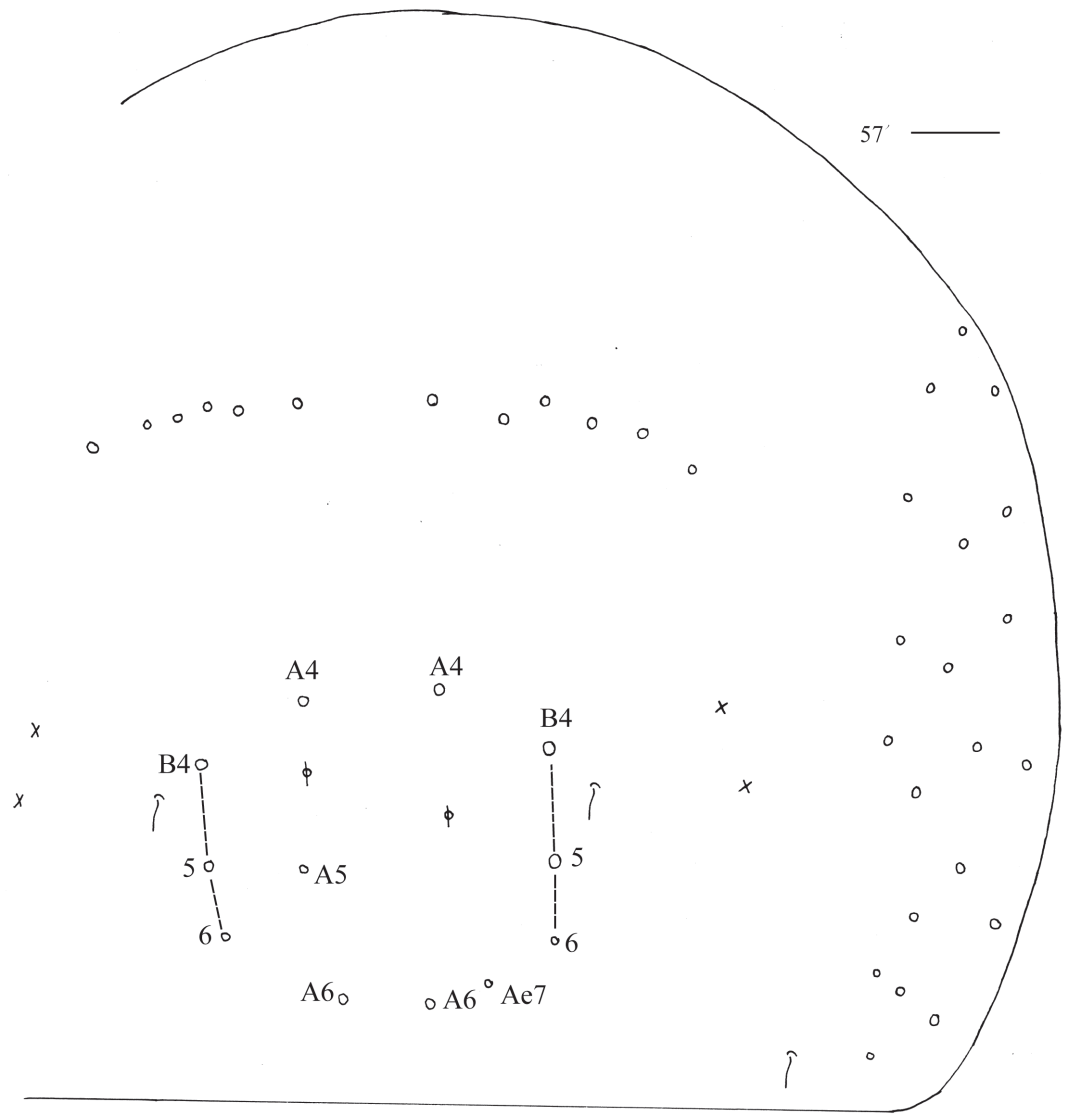
Chaetotaxy of Abd. IV of *Homidiachangensis* sp. nov. Scale bar: 50 μm.

**Figures 58–64.**
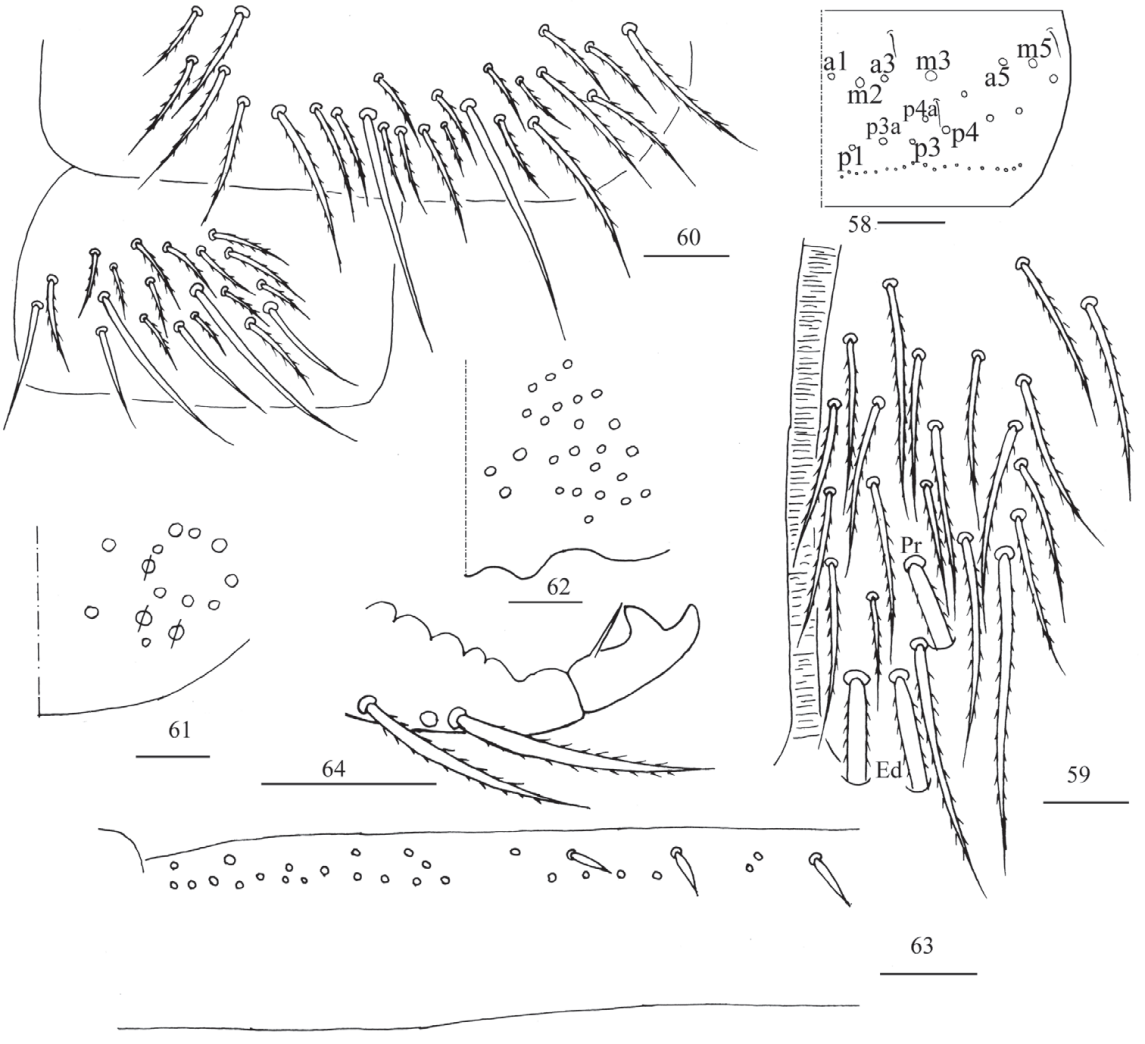
*Homidiachangensis* sp. nov. **58** chaetotaxy of Abd. V **59** anterior face of ventral tube **60** posterior face and lateral flap of ventral tube **61** manubrial plaque (a circle with a slash means a pseudopore) **62** ventro-apical part of manubrium **63** proximal section of dens (circles also representing spines) **64** mucro. Scale bars: 20 μm.

***Abdomen***. Range of Abd. IV length as 6.03–10.40× as dorsal axial length of Abd. III. Abd. I with 11 (a1a, a1–3, a5, m2–4, m2i, m4i, m4p) mac, ms antero-external to sens. Abd. II with six (a2, a3, m3, m3e, m3ea, m3ep) central, one (m5) lateral mac and two sens. Abd. III with two (a2, m3) central and four (am6, pm6, m7a, p6) lateral mac, one ms and two sens (Fig. 56). Abd. IV with two normal sens; anteriorly with six or seven mac arranged in irregular transverse row, posteriorly with 5–7 (A4, A6, B4–6, A5 and Ae7 sometimes present) central mac, laterally with 20–22 mac (Fig. 57). Abd. V with three sens, middle one posterior to m3 (Fig. 58). Anterior face of ventral tube with 24–27 ciliate chaetae, 3+3 of them as mac, line connecting proximal (Pr) and external-distal (Ed) mac oblique to median furrow (Fig. 59); posterior face with two or four distal smooth and numerous ciliate chaetae; lateral flap with six smooth and 14–16 ciliate chaetae (Fig. 60). Manubrial plaque dorsally with 11 or 12 ciliate chaetae and 2–4 pseudopores (Fig. 61); ventrally with 25–28 ciliate chaetae on each side (Fig. 62). Dens with 16–28 smooth inner spines (Fig. 63). Mucro bidentate with subapical tooth larger than apical one; tip of basal spine reaching apex of subapical tooth; distal smooth section of dens shorter than mucro in length (Fig. 64).

#### Ecology.

In the leaves litter of *Phyllostachysedulis*.

#### Etymology.

Named after its locality: Nanchang City, which is abbreviated as Chang.

#### Remarks.

The new species is characterised by its colour pattern and coxal macrochaetotaxy, and can be easily distinguished from all known species of *Homidia*. It is similar to the Chinese species *H.huashanensis* Jia, Chen & Christiansen, 2005, *H.jordanai* Pan, Shi & Zhang, 2011, and *H.unichaeta* Pan, Shi & Zhang, 2010 and the Korean species *H.koreana* Lee & Lee, 1981 in colour pattern, but significant differences exist between these species, such as chaetotaxy on Abd. I and IV and number of dental spines (Table 3).

**Table 3.** Comparison between *H.changensis* sp. nov. and similar species.

Characters	*H.changensis* sp. nov.	*H.huashanensis*	*H.jordanai*	*H.unichaeta*	*H.koreana*
Ground colour	yellow	hazel	pale yellow	pale to yellowish	brown
Length ratio of antenna to body	0.50–0.57	0.67	1.00	0.80–1.00	unknown
Chaetal formula of labial base	M_1_M_2_ReL_1_L_2_	MRE(e)L_1_L_2_	MReL_1_L_2_	MRel_1_L_2_	MReL_1_L_2_
Chaetae a1, a1a on Abd. I	present	present	absent	a1 rarely present, a1a absent	absent
Central mac on Abd. III	2	2	1	2	2
Centro-posterior mac on Abd. IV	5–7	7–9	2(3)	1	6
Dental spines	16–28	80–114	20–40	19–23	40–50

### ﻿ Homidia linhaiensis

Taxon classificationAnimaliaCollembolaEntomobryidae

Shi, Pan & Qi, 2009

EAAE3670-74D0-5B57-9646-2C861250BBD9

Figs 65–66 , 67, 68 , 69 , 70–75

Homidia linhaiensis Shi, Pan & Qi, 2009: 63.

#### Examined specimens.

2♀ on slides, **China**, Jiangxi Province, Pingxiang City, Luxi Town, Shankouyan Park, 27°36'55"N, 114°01'39"E, 144 m asl, sample number 1231, collected by Y-T Ma, 7-XI-2020, in the rotten leaves of *Salixbabylonica*; 5♀ on slides, **China**, Jiangxi Province, Nanchang City, Xinjian District, Shizifeng Park, 28°48'48"N, 115°43'15"E, 193 m asl, sample number 1241, collected by Y-T Ma, 12-XI-2020, in the leaves litter of *Phyllostachysedulis*; 2♀ on slides, **China**, Jiangxi Province, Shangrao City, Yunbifeng Park, 28°27'47"N, 117°58'55"E, 101 m asl, sample number 1246, collected by Y-T Ma, 14-XI-2020, in the leaves litter of *Phyllostachysedulis*.

#### Description.

***Size*.** Body length up to 2.00 mm.

**Figures 65, 66.**
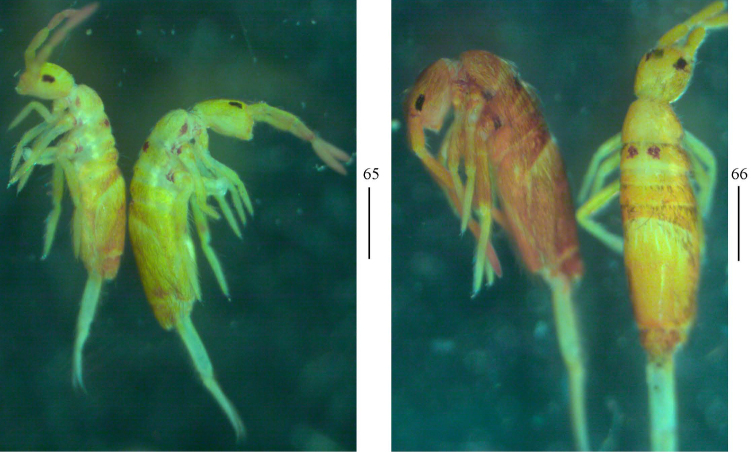
Habitus of *Homidialinhaiensis*. Scale bars: 500 μm.

***Colouration*.** Ground colour yellow. Ant. III & IV with scattered blue pigment. Eye patches dark blue. Th. III with a pair of dark blue spots and coxae and lateral of Th. II also with blue pigment (Figs 65, 66).

**Figures 67, 68.**
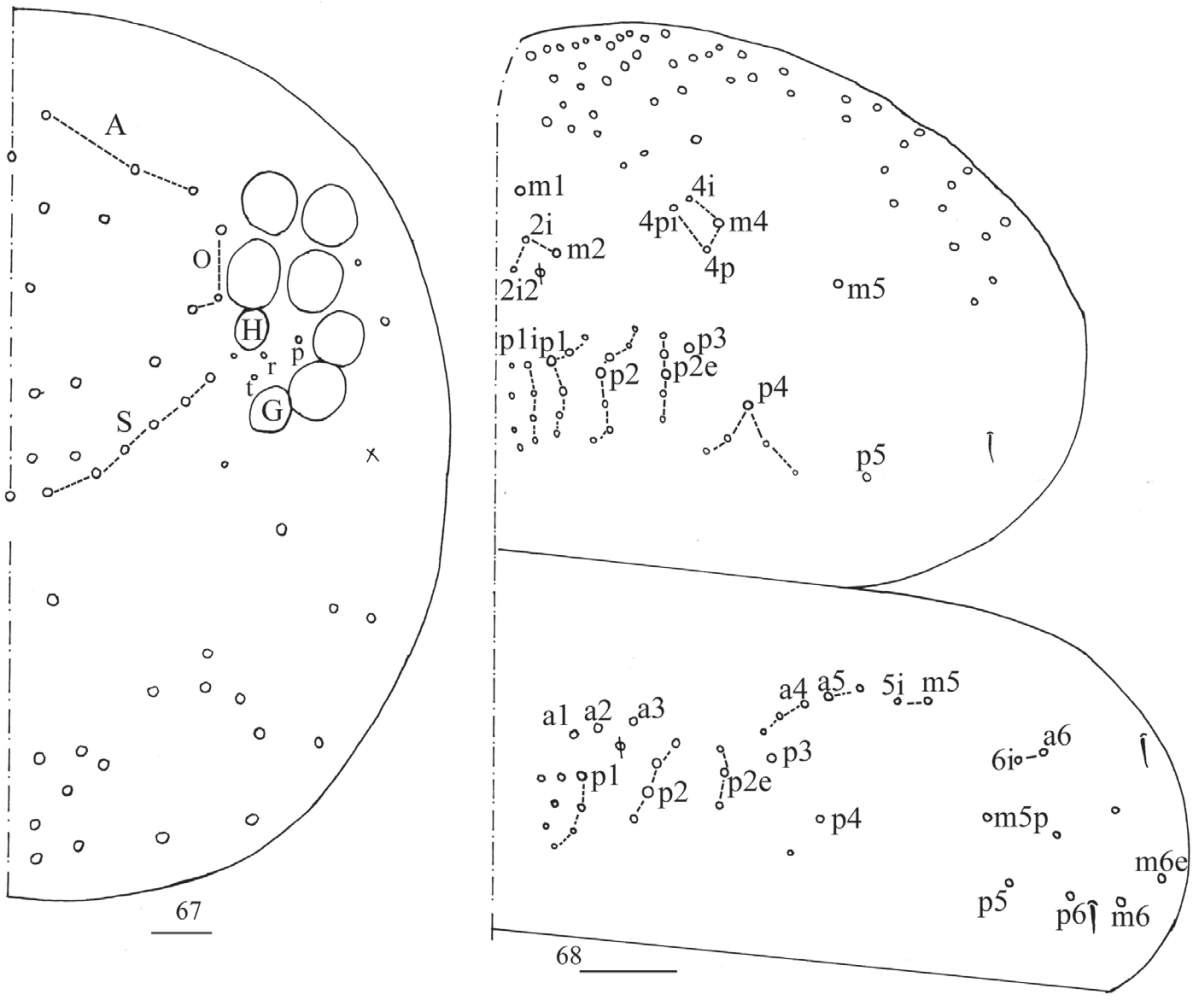
*Homidialinhaiensis***67** dorsal chaetotaxy of head **68** chaetotaxy of Th. II–III. Scale bars: 20 μm (**67)**; 50 μm (**68**).

***Head*.** Antenna 0.44–0.59× body length; antennal segment ratio I: II: III: IV = 1: 1.30–1.47: 1.21–1.33: 2.13–2.51. Eyes 8 + 8, interocular chaetae with p, r, and t. Dorsal cephalic chaetotaxy with three antennal (A), three ocellar (O) and six sutural (S) mac (Fig. 67). Chaetal formula of labial base as MRel_1_L_2_, chaetae e and l_1_ smooth, others ciliate, R/M as 0.56.

***Thorax*.**Th. II with four medio-medial (m1, m2, m2i, m2i2), four medio-sublateral (m4, m4i, m4p, m4pi), 33 posterior mac. Th. III with 36–39 mac and two sens (Fig. 68).

**Figure 69.**
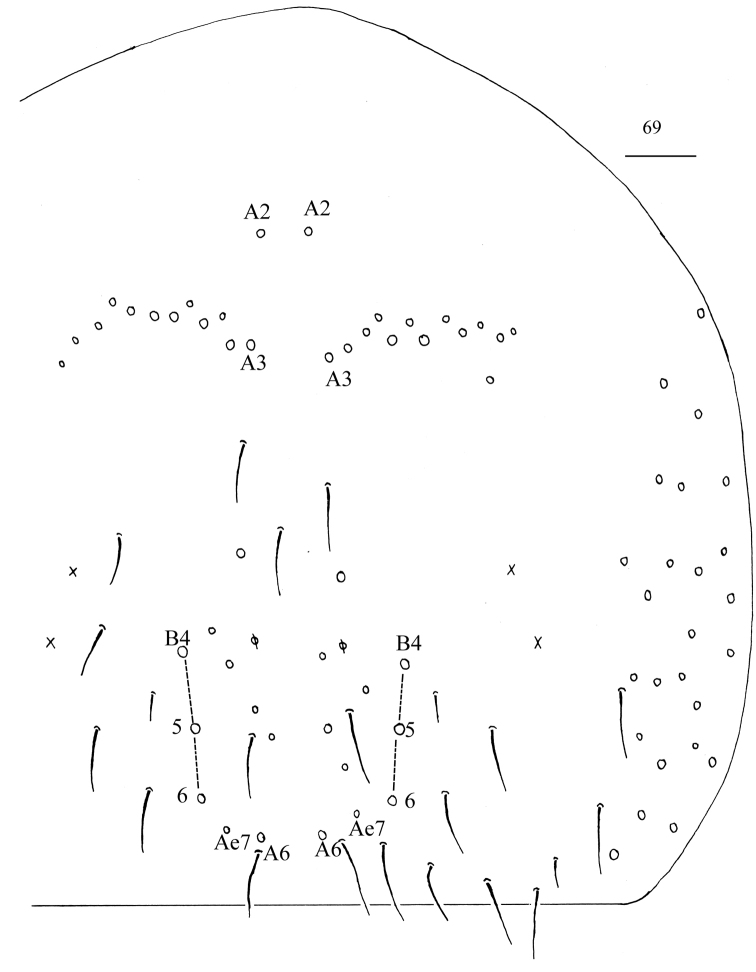
Chaetotaxy of Abd. IV of *Homidialinhaiensis*. Scale bar: 50 μm.

**Figures 70–75.**
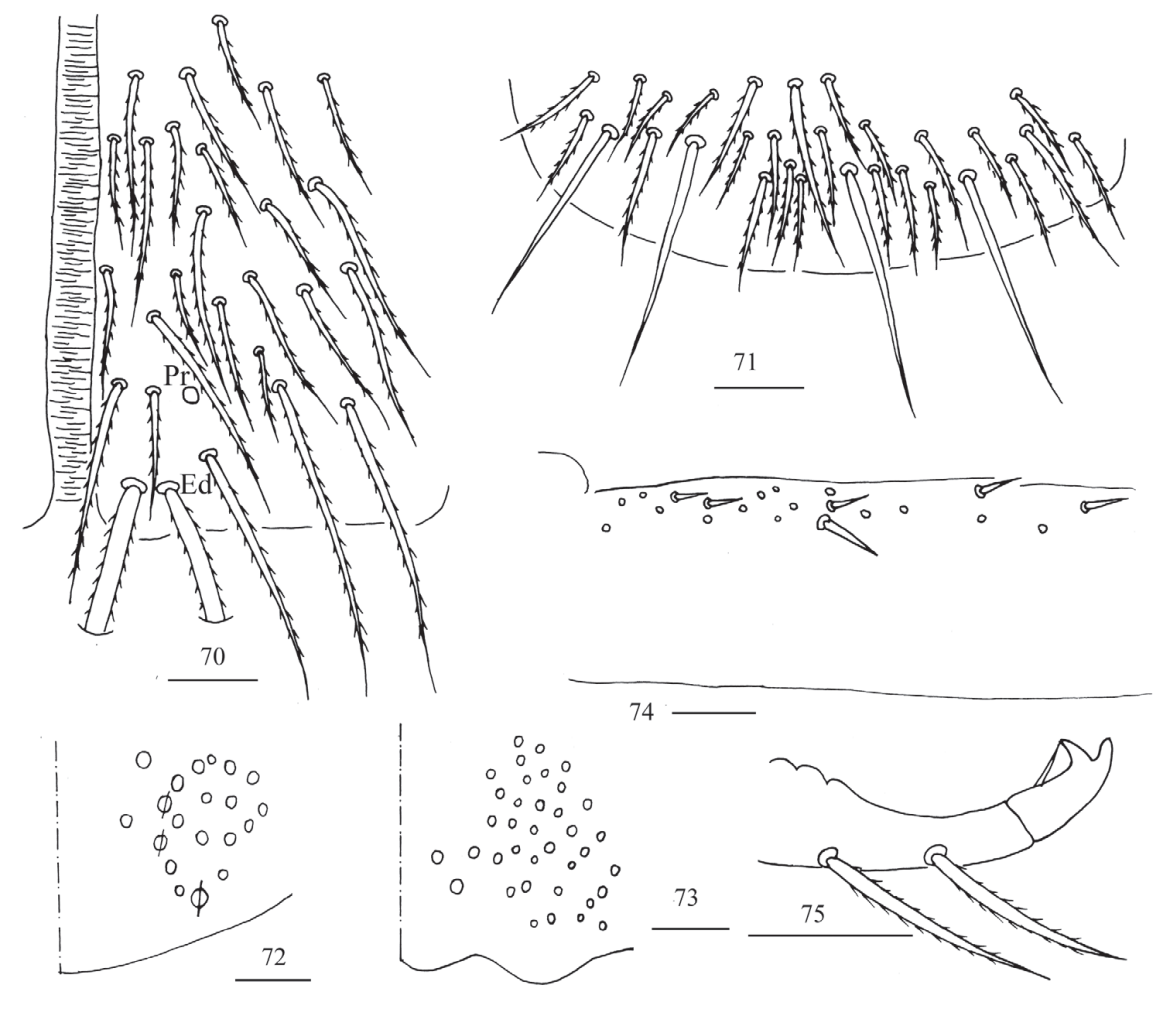
*Homidialinhaiensis***70** anterior face of ventral tube **71** posterior face of ventral tube **72** manubrial plaque **73** ventro-apical part of manubrium **74** proximal section of dens (circles also representing spines) **75** mucro. Scale bars: 20 μm.

***Abdomen*.** Range of Abd. IV length as 6.28–9.32× as dorsal axial length of Abd. III. Abd. I with 10 (a2, a3, a5, m2–5, m2i, m4i, m4p) mac, ms antero-internal to sens. Abd. II with six (a2, a3, m3, m3e, m3ea, m3ep) central, one (m5) lateral mac and two sens. Abd. III with two (a2, m3) central and five (am6, pm6, m7a, p6, p7) lateral mac, one ms and two sens. Abd. IV anteriorly with 9–13 mac arranged in irregular transverse row, A2 always present and anterior to transverse row; posteriorly with 10–16 central mac, laterally with 23–27 mac (Fig. 69). Anterior face of ventral tube with 24–28 ciliate chaetae, 3+3 of them as mac, line connecting proximal (Pr) and external-distal (Ed) mac oblique to median furrow (Fig. 70); posterior face with four distal smooth and numerous ciliate chaetae (Fig. 71). Manubrial plaque dorsally with 14–17 ciliate chaetae and three pseudopores (Fig. 72); ventrally with 37 ciliate chaetae on each side (Fig. 73). Dens with 12–21 smooth inner spines (Fig. 74). Mucro bidentate with subapical tooth larger than apical one; tip of basal spine reaching apex of subapical tooth; distal smooth section of dens almost equal to than mucro in length (Fig. 75).

#### Remarks.

This species was first described from Zhejiang Province by Shi et al. (2009) and can be easily distinguished from other known species of the genus by two small blue spots on Th. III, five mac on Abd. III laterally, presence of A2 on Abd. IV. The characters of our specimens agree well with the original description in chaetotaxy of body, labium, colour pattern, and other characters, but there are five smooth chaetae on posterior face of ventral tube from Zhejiang and four smooth chaetae from that from Jiangxi. In fact, the number of smooth chaetae on posterior face of ventral tube may varies intraspecifically from two to five in some species of the genus. Chaetotaxy of manubrial plaque is added here.

#### Distribution.

China (Jiangxi, Zhejiang).

### ﻿ Homidia socia

Taxon classificationAnimaliaCollembolaEntomobryidae

Denis, 1929

313CE085-0E01-5C0C-8ED4-1DED816B8A5E

Figs 76 , 77, 78 , 79 , 80 , 81–86

Homidia socia Denis, 1929: 310.

#### Examined specimens.

3♀ on slides, **China**, Jiangxi Province, Nanchang City, Xinjian District, Jiuxi, 28°47'56"N, 115°45'11"E, 168 m asl, sample number 1243, collected by Y-T Ma, 12-XI-2020.

#### Description.

***Size*.** Body length up to 2.16 mm.

**Figure 76.**
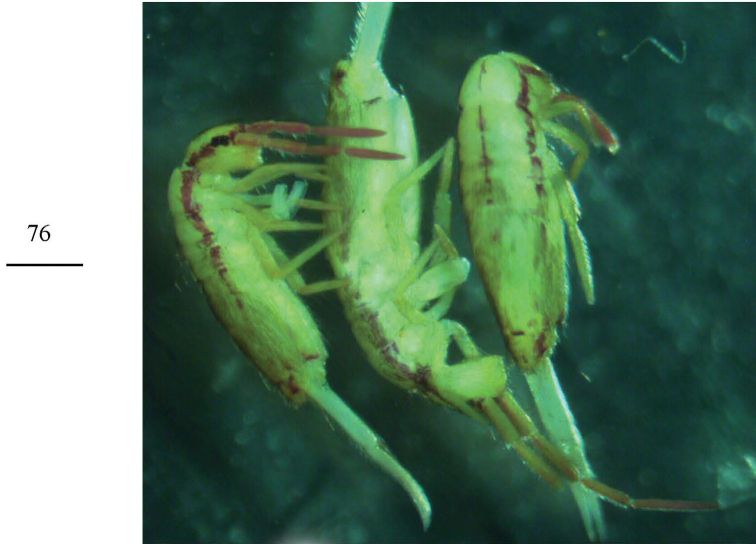
Habitus of *Homidiasocia* Scale bar: 500 μm.

***Colouration*.** Ground ground colour pale yellow. Ant. I–IV with scattered blue pigment. Eye patches dark blue. A pair of longitudinal blue stripes present along lateral side of head to Abd. III. Medial longitudinal narrow stripe present from Th. II to Abd. III. Abd. V with blue pigment (Fig. 76).

**Figures 77, 78.**
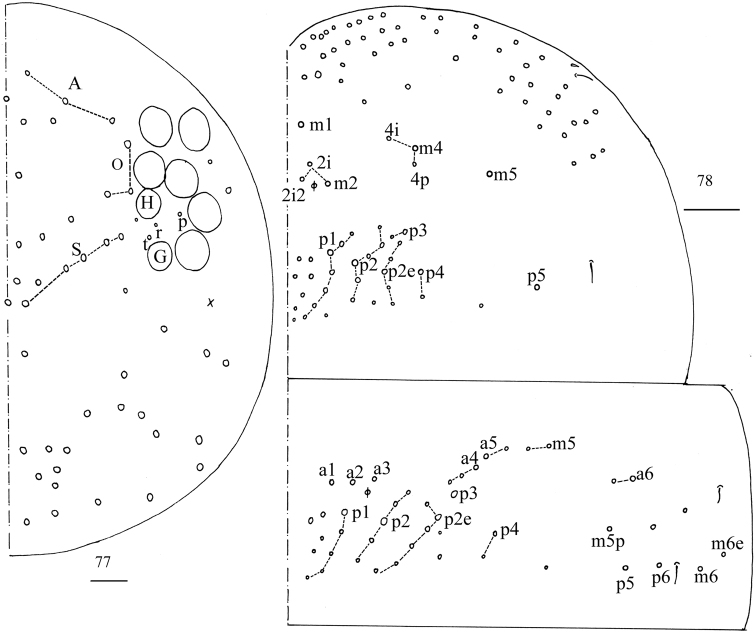
*Homidiasocia***77** dorsal chaetotaxy of head **78** chaetotaxy of Th. II–III. Scale bars: 20 μm (**77**); 50 μm (**78**).

***Head*.** Antenna 0.52–0.66× body length; antennal segment ratio I: II: III: IV = 1: 1.28–1.40: 1.00–1.20: 1.67–2.11. Eyes 8 + 8, G and H smaller than others, interocular chaetae with p, r, and t. Dorsal cephalic chaetotaxy with three antennal (A), three ocellar (O) and five sutural (S) mac (Fig. 77). Chaetal formula of labial base as MREL1L2, all ciliate, R/M as 0.67–0.72.

***Thorax*.**Th. II with four medio-medial (m1, m2, m2i, m2i2), three medio-sublateral (m4, m4i, m4p), 30–33 (24) posterior mac. Th. III with 41–45 mac and two sens (Fig. 78). Pseudopores on coxa I–III as 2, 3, 2, respectively; coxal macrochaetal formula as 3/4+3, 3/4+2. Trochanteral organ with 39 smooth chaetae. Tenent hairs clavate and almost equal to inner edge of unguis. Unguis with four inner teeth, basal pair located at 0.38–0.45 distance from base of inner edge of unguis, distal unpaired teeth at 0.65–0.67 and 0.80–0.85 distance from base; unguiculus lanceolate, outer edge slightly serrate.

**Figure 79.**
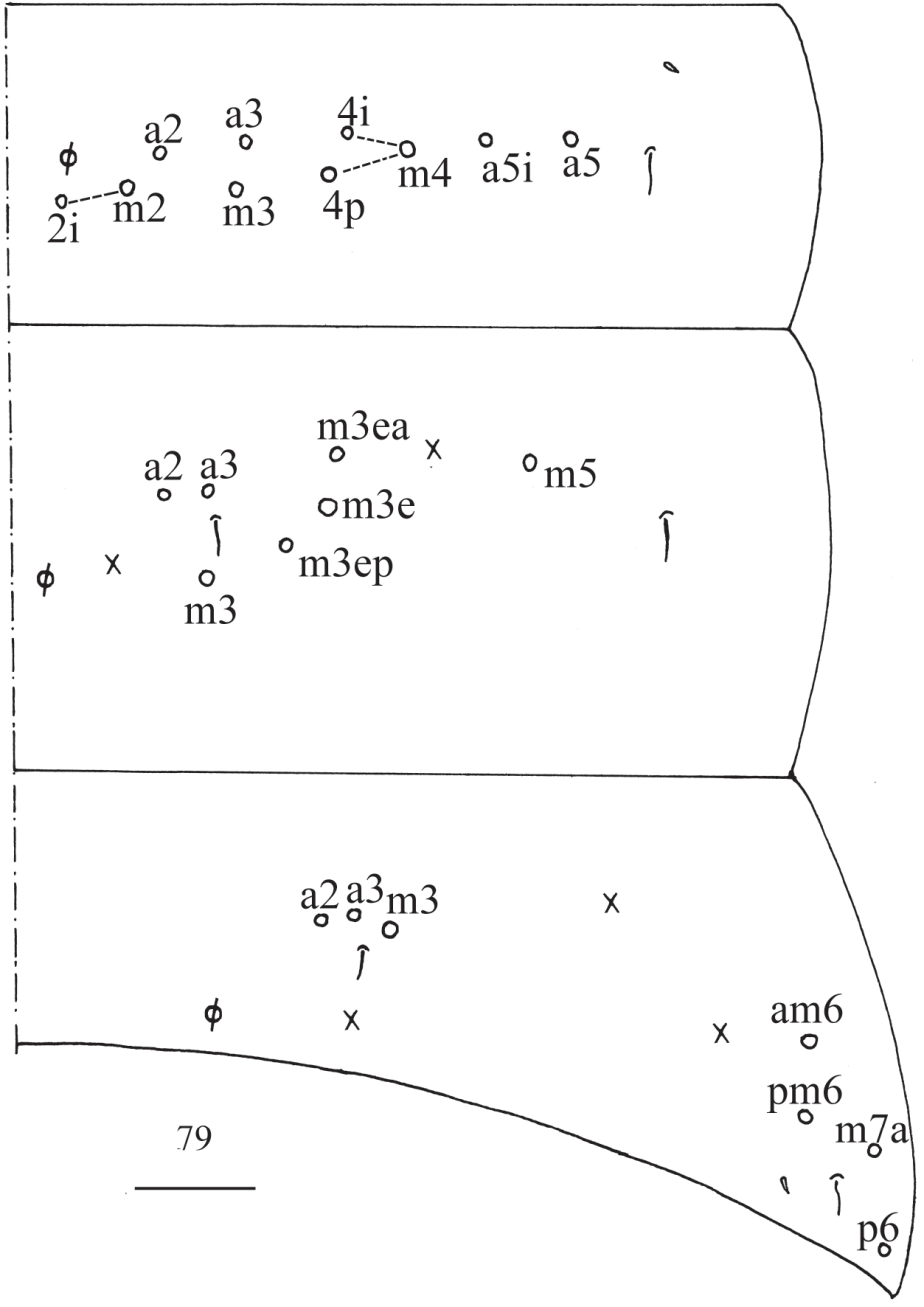
Chaetotaxy of Abd. I–III of *Homidiasocia*. Scale bar: 50 μm.

**Figure 80.**
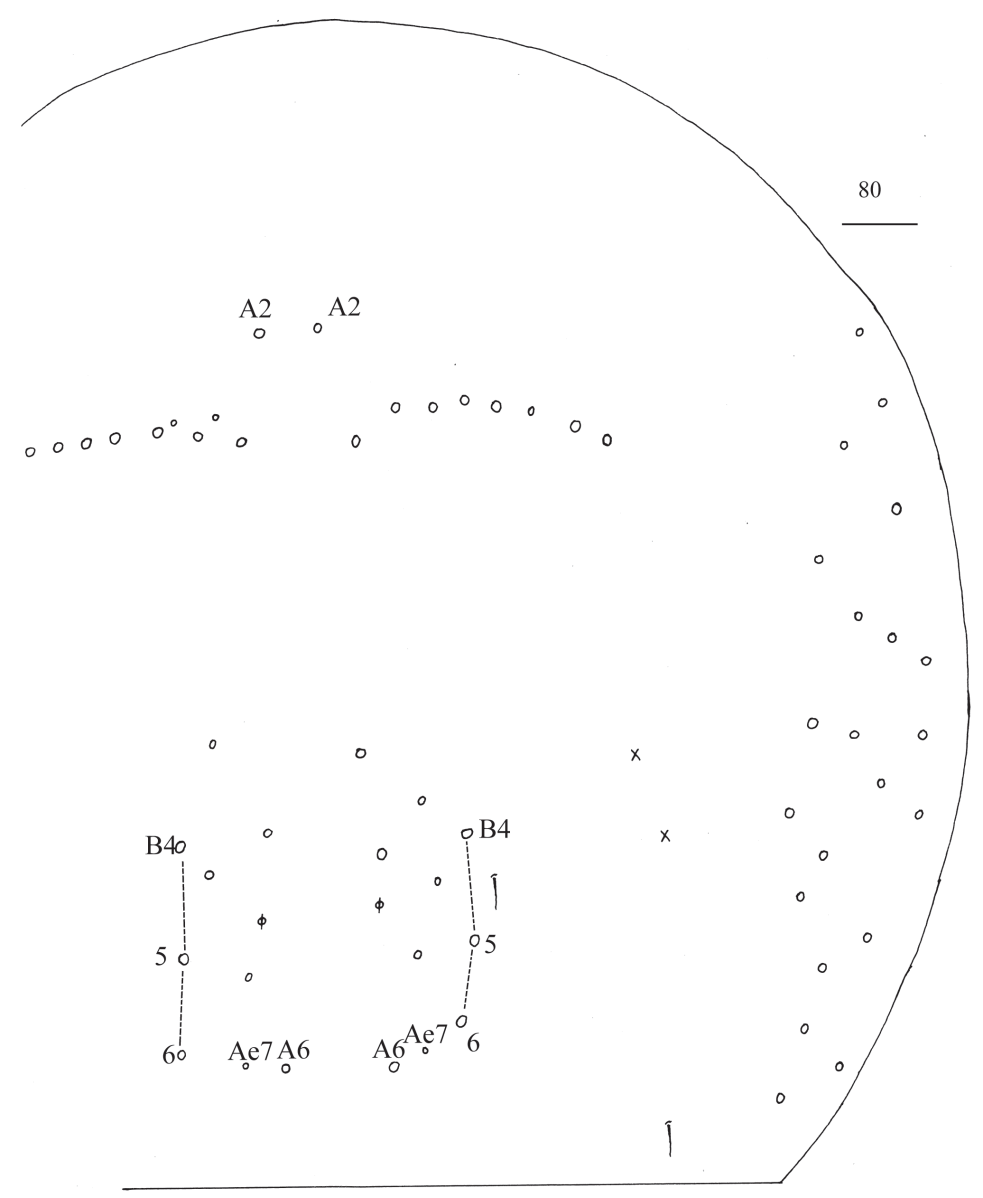
Chaetotaxy of Abd. IV of *Homidiasocia*. Scale bar: 50 μm.

**Figures 81–86.**
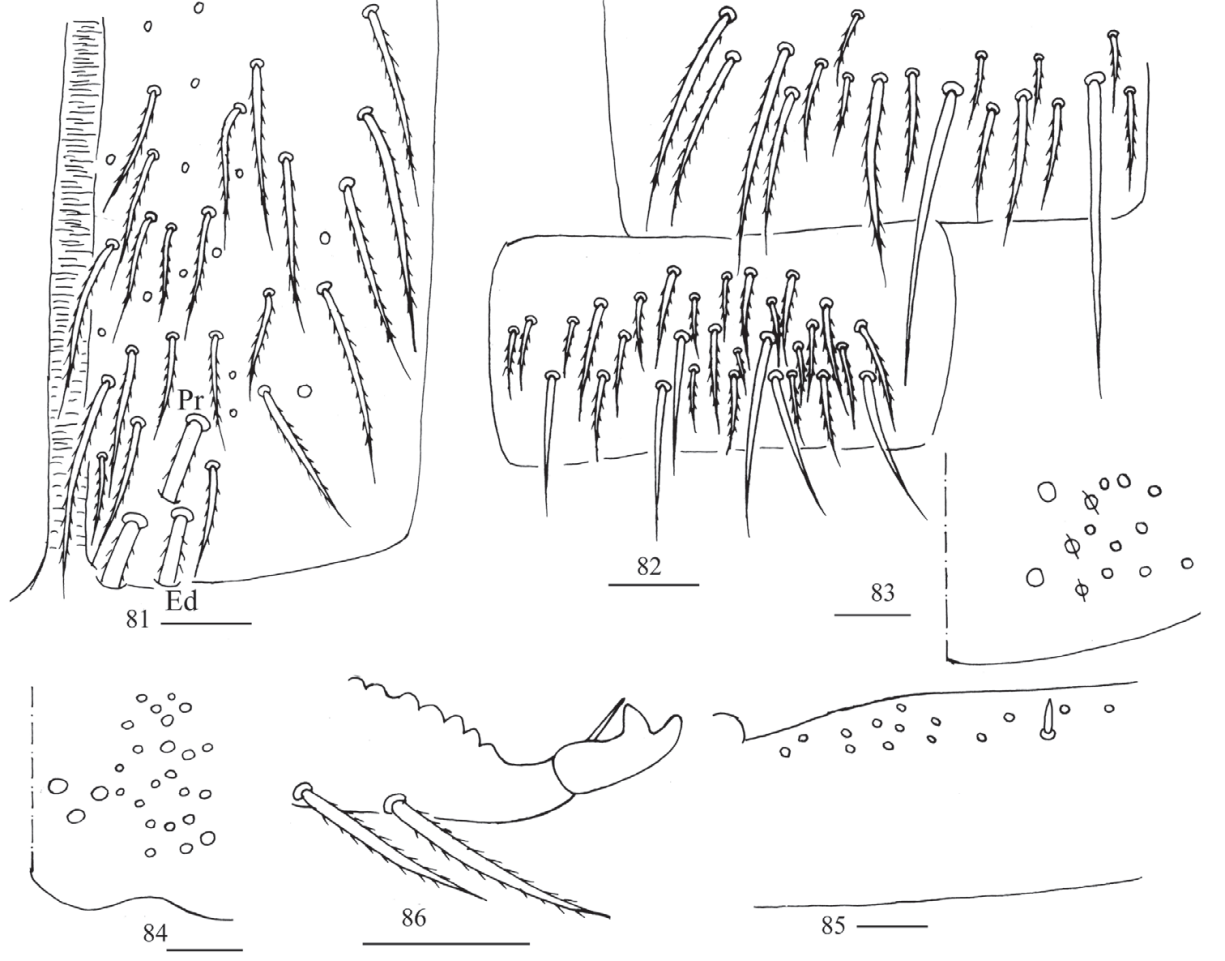
*Homidiasocia***81** anterior face of ventral tube **82** posterior face and lateral flap of ventral tube **83** manubrial plaque **84** ventro-apical part of manubrium **85** proximal section of dens (circles also representing spines) **86** mucro. Scale bars: 20 μm.

***Abdomen*.** Range of Abd. IV length as 6.25–10.12× as dorsal axial length of Abd. III. Abd. I with 10 (a2, a3, a5, a5i, m2–5, m2i, m4i, m4p) mac, ms antero-external to sens. Abd. II with six (a2, a3, m3, m3e, m3ea, m3ep) central, one (m5) lateral mac, and two sens. Abd. III with two or three (a2, m3, a3 sometimes absent) central and four (am6, pm6, m7a, p6) lateral mac, one ms, and two sens (Fig. 79). Abd. IV anteriorly with eight or nine mac arranged in irregular transverse row, A2 always present and anterior to transverse row; posteriorly with 6–10 central mac; laterally with 18–22 mac (Fig. 80). Anterior face of ventral tube with 39 ciliate chaetae, 3+3 of them as mac, line connecting proximal (Pr) and external-distal (Ed) mac oblique to median furrow (Fig. 81); posterior face with two distal smooth and numerous ciliate chaetae; lateral flap with six smooth and 24 ciliate chaetae (Fig. 82). Manubrial plaque dorsally with 11–14 ciliate chaetae and three pseudopores (Fig. 83); ventrally with 26 ciliate chaetae on each side (Fig. 84). Dens with 15 smooth inner spines (Fig. 85). Mucro bidentate with subapical tooth larger than apical one; tip of basal spine reaching apex of subapical tooth; distal smooth section of dens shorter than mucro in length (Fig. 86).

#### Ecology.

In the litter of leaves of *Phyllostachysedulis*.

#### Remarks.

This species was first described from Fujian Province, China by Denis (1929), mainly based on its colour pattern with three longitudinal stripes on dorsal side. Yosii (1942), Stach (1965), Lee and Park (1989), and Chiristiansen and Bellinger (1980, 1992) reported it from Japan, Vietnam, Taiwan (China), and USA, respectively, and their descriptions were relatively simple. Jordana (2012) also reported it based on Vietnamese and Japanese specimens that corresponded in colour pattern to H.sociaformaflava Yosii, 1953 from Japan. In this work, several characters not previously mentioned are added, such as the chaetotaxy of the head, ventral tube, and manubrial plaque. Differences exist between the specimens collected from Jiangxi Province and other authors’ previous descriptions that are listed in Table 4. Our specimens, with 15 spines on dens, are similar to those examined by Jordana (2012) which had 14 spines. Up to 30 spines were given by Denis (1929) for the type specimens of large size from Fujian. These specimens or specimens from the type locality will therefore need to be redescribed to confirm the assignment of our specimens as well as those described by Jordana (2012) to *H.socia*.

**Table 4.** Comparison of *H.socia* between different descriptions.

Characters	This work	Denis (1929)	Christiansen and Bellinger (1980, 1992)	Stach (1965)	Jordana (2012)
Chaetal formula of labial base	MREL_1_L_2_	unknown	MREL_1_L_2_	unknown	unknown
Mac on Abd. I	10	unknown	11 or 15	9	unknown
Central mac on Abd. II	6	unknown	5–6	6	6
Central mac on Abd. III	2–3	unknown	3	3	3
Mac A2 on Abd. IV	present	unknown	present	unknown	present
Mac of transverse row on Abd. IV	8–9	unknown	8	8	7
Centro-posterior mac on Abd. IV	6–10	unknown	5	8	5
Dental spines	15	up to 30	<20	7 or 11	14*

* Jordana (2012) gave 14 spines on dens on the specimens of Vietnam and Japan he examined, and 14–30 spines in the species redescription that included the values given by Denis (1929) in the original description.

#### Distribution.

Japan, USA, Vietnam, and China (Anhui, Fujian, Guangxi, Jiangsu, Jiangxi, Taiwan, Zhejiang).

## ﻿Discussion

The genus contains 75 known species that are distributed in the U.S.A. and the eastern part of Asia, especially China, Korea, and Japan (Table 5). They usually live in coastal areas from tropical to temperate zones, maybe because humidity is high in these regions. Most species of the genus are endemic (Table 5). However, some of them, such as *H.sauteri* and *H.socia*, are widespread, and were reported from China, Japan, Korea, and the U.S.A. Jia et al. (2005) pointed out that these two species are so widely distributed that they may have been transported by human activity. This hypothesis would, however, need more evidence.

**Table 5.** Distribution records of *Homidia* species around the world.

Species	China	Japan	Korea	India	Indonesia & Singapore	Vietnam	USA
*H.acutus* sp. nov.	√						
*H.allospila* (Börner, 1909)		√					
*H.amethystinoides* Jordana & Baquero, 2010		√					
*H.anhuiensis* Li & Chen, 1997	√						
*H.apigmenta* Shi, Pan & Zhang, 2010	√						
*H.breviseta* Pan, 2022	√						
*H.changensis* sp. nov.	√						
*H.chosonica* Szeptycki, 1973			√				
*H.chroma* Pan & Yang, 2019	√						
*H.chrysothrix* Yosii, 1942		√					
*H.cingula* (Börner, 1906)					√		
*H.dianbaiensis* (Lin, 1985)	√						
*H.emeiensis* Jia, Chen & Christiansen, 2004	√						
*H.fascia* Wang & Chen, 2001	√						
*H.flava* Yosii, 1953		√					
*H.flavonigra* Szeptycki, 1973			√				
*H.formosana* Uchida, 1943	√						
*H.fujiyamai* Uchida, 1954		√					
*H.glassa* Nguyen, 2001						√	
*H.grisea* Lee & Lee, 1981			√				
*H.haikea* Christiansen & Bellinger, 1992							√
*H.hangzhouensis* Pan & Ma, 2021	√						
*H.heugsanica* Lee & Park, 1984			√				
*H.hexaseta* Pan, Shi & Zhang, 2011	√						
*H.hihiu* Christiansen & Bellinger, 1992							√
*H.hjesanica* Szeptycki, 1973			√				
*H.huashanensis* Jia, Chen & Christiansen, 2005	√						
*H.insularis* (Carpenter, 1904)							√
*H.jordanai* Pan, Shi & Zhang, 2011	√						
*H.kali* (Imms, 1912)				√			
*H.koreana* Lee & Lee, 1981			√				
*H.laha* Christiansen & Bellinger, 1992	√						√
*H.lakhanpurii* Baquero & Jordana, 2015				√		√	
*H.latifolia* Chen & Li, 1999	√						
*H.leei* Chen & Li, 1997	√						
*H.leniseta* Pan & Yang, 2019	√						
*H.linhaiensis* Shi, Pan & Qi, 2009	√						
*H.maijiensis* Zhou & Ma, 2022	√						
*H.mediaseta* Lee & Lee, 1981			√				
*H.mediofascia* Shi, Pan & Bai, 2009	√						
*H.minuta* Kim & Lee, 1995			√				
*H.multidentata* Nguyen, 2005						√	
*H.munda* Yosii, 1956		√	√				
*H.nigra* Lee & Lee, 1981			√				
*H.nigrifascia* Ma & Pan, 2017	√						
*H.nigrocephala* Uchida, 1943	√	√					
*H.obliquistria* Ma & Pan, 2017	√						
*H.pentachaeta* Li & Christiansen, 1997	√						
*H.phjongjangica* Szeptycki, 1973	√		√				
*H.polyseta* Chen, 1998	√						
*H.pseudofascia* Pan, Zhang & Li, 2015	√						
*H.pseudoformosana* Kang & Park, 2012			√				
*H.pseudosinensis* Shi & Pan, 2012	√						
*H.qimenensis* Yi & Chen, 1999	√						
*H.quadrimaculata* Pan, 2015	√						
*H.quadriseta* Pan, 2018	√						
*H.rosannae* Jordana & Baquero, 2010		√					
*H.sauteri* (Börner, 1909)	√	√	√				√
*H.sichuanensis* Jia, Zhang & Jordana, 2010	√						
*H.similis* Szeptycki, 1973	√		√				
*H.sinensis* Denis, 1929	√	√				√	
*H.socia* Denis, 1929	√	√				√	√
*H.sotoi* Jordana & Baquero, 2010		√					
*H.speciosa* Szeptycki, 1973			√				
*H.subcingula* Denis, 1948						√	
*H.taibaiensis* Yuan & Pan, 2013	√						
*H.tiantaiensis* Chen & Lin, 1998	√						
*H.tibetensis* Chen & Zhong, 1998	√						
*H.transitoria* Denis, 1929	√						
*H.triangulimacula* Pan & Shi, 2015	√						
*H.unichaeta* Pan, Shi & Zhang, 2010	√					√	
*H.wanensis* Pan & Ma, 2021	√						
*H.xianjuensis* Wu & Pan, 2016	√						
*H.yandangensis* Pan, 2015	√						
*H.yosiii* Jordana & Baquero, 2010		√					
*H.zhangi* Pan & Shi, 2012	√						
*H.ziguiensis* Jia, Chen & Christiansen, 2003	√						

Most species of *Homidia* are heavily pigmented and their colour patterns vary only slightly among specimens of the same species, so colour pattern appears to be a significant character for the morphological taxonomy of the genus. However, colour pattern may exhibit some variability between some species, such as *H.fascia* Wang & Chen, 2001 and *H.pseudofascia* Pan, Zhang & Li, 2015. The new species, *H.acutus* sp. nov. described here shares almost the same colour pattern as *H.zhangi*, but the differences in other characters are significant.

### ﻿Key to the Chinese species of *Homidia*

**Table:** 

1	Mental chaetae expanded or leaf-like	**2**
–	Mental chaetae normal ciliate	**9**
2	Body without obvious colour pattern except eye patches	***H.apigmenta***
–	Body with obvious colour pattern except eye patches	**3**
3	Abd. I–III laterally with oblique stripes	***H.obliquistria***
–	Abd. I–III laterally without oblique stripes	**4**
4	Abd. IV with mac A2	***H.ziguiensis***
–	Abd. IV without mac A2	**5**
5	Central Abd. IV with roughly Y-shaped patch	***H.qimenensis***
–	Abd. IV without Y-shaped patch	**6**
6	Abd. IV anteriorly with an interrupted dark transverse stripe	**7**
–	Abd. IV anteriorly without dark transverse stripe	**8**
7	Abd. IV anteriorly with 4–7 mac on each side	***H.latifolia***
–	Abd. IV anteriorly with 22–24 mac on each side	***H.polyseta***
8	Labial basal chaetae L_1_ and L_2_ expanded	***H.triangulimacula***
–	Labial basal chaetae L_1_ and L_2_ unexpanded	***H.wanensis***
9	Body without obvious colour pattern except eye patches	**10**
–	Body with obvious colour pattern except eye patches	**12**
10	Labial basal chaeta L_1_ ciliate, Abd. III without mac a2	***H.jordanai***
–	Labial basal chaeta L_1_ smooth, Abd. III with mac a2	**11**
11	Abd. IV anteriorly with 3–8 mac and posteriorly 1 mac on each side	***H.unichaeta***
–	Abd. IV anteriorly with 10–12 mac and posteriorly 2 mac on each side	***H.tibetensis***
12	Head entirely dark	**13**
–	Head not entirely dark	**15**
13	Abd. IV anteriorly with a transverse stripe	***H.nigrocephala***
–	Abd. IV anteriorly without transverse stripe	**14**
14	Th. II–III entirely dark	***H.anhuiensis***
–	Th. II–III with slightly brown pigment	***H.taibaiensis***
15	Abd. IV with mac A2	.**16**
–	Abd. IV without mac A2	**18**
16	Labial basal chaetae E & L_1_ ciliate	***H.socia***
–	Labial basal chaetae e & l_1_ smooth	**17**
17	Abd. III laterally with 5 mac	***H.linhaiensis***
–	Abd. III laterally with 4 mac	***H.tiantaiensis***
18	Abd. IV almost entirely dark or with uniform colour	**19**
–	Abd. IV with some colour patterns	**25**
19	Abd. IV almost entirely dark	***H.emeiensis***
–	Abd. IV not entirely dark	**20**
20	Abd. III laterally with 5 chaetae	***H.pentachaeta***
–	Abd. III laterally with 4 chaetae	**21**
21	Tenent hairs pointed	***H.acutus* sp. nov.**
–	Tenent hairs clavate	**22**
22	Th. II–III medially with a longitudinal stripe	***H.yandangensis***
–	Th. II–III medially without a longitudinal stripe	**23**
23	Th. III without mac p4, labial chaeta L_1_ smooth	***H.zhangi***
–	Th. III with mac p4, labial chaeta L_1_ ciliate	**24**
24	Ground colour hazel, dens with 80–114 spines	***H.huashanensis***
–	Ground colour yellow, dens with 16–28 spines	***H.changensis* sp. nov**.
25	Abd. III without obvious colour pattern	**26**
–	Abd. III with obvious colour pattern	**34**
26	Th. II medially with colour pattern	**27**
–	Th. II medially without colour pattern	**29**
27	Th. II medially with a longitudinal stripe	***H.mediofascia***
–	Th. II medially with a pair of stripes	**28**
28	Th. III with a pair of patches	.***H.fascia***
–	Th. III without a pair of patches	***H.pseudofascia***
29	Abd. IV anteriorly with obvious colour pattern	**30**
–	Abd. IV anteriorly without obvious colour pattern	**32**
30	Mac a2 on Abd. III absent	***H.formosana***
–	Mac a2 on Abd. III present	**31**
31	Head with 8 sutural mac	***H.hangzhouensis***
–	Head with 9 sutural mac	***H.hexaseta***
32	Ground colour pale yellow	***H.dianbaiensis***
–	Ground colour not pale yellow	.**33**
33	Labial basal chaeta E ciliate	***H.maijiensis***
–	Labial basal chaeta e smooth	***H.phjongjangica***
34	Abd. IV anteriorly with 2 mac on each side	**35**
–	Abd. IV anteriorly with more than 2 mac on each side	**36**
35	Labial chaetae l_1_ and l_2_ smooth	***H.leniseta***
–	Labial chaetae l_1_ and l_2_ ciliate	***H.quadriseta***
36	Abd. IV posteriorly with 9–11 mac on each side	***H.xianjuensis***
–	Abd. IV posteriorly with less than 9 mac on each side	**37**
37	Th. III dorsally without obvious colour pattern	**38**
–	Th. III dorsally with obvious colour pattern	**40**
38	Th. II medially with colour pattern, Abd. II entirely dark	***H.nigrifascia***
–	Th. II medially without colour pattern, Abd. II not entirely dark	**39**
39	Unguis with 3 inner teeth	***H.chroma***
–	Unguis with 4 inner teeth	. ***H.laha***
40	Th. II medially without colour pattern	**41**
–	Th. II medially with colour pattern	**45**
41	Transverse band of Th. III not reaching lateral edge of body	**42**
–	Transverse band of Th. III reaching lateral edge of body	**43**
42	Abd. I with 14 mac	***H.breviseta***
–	Abd. I with 9 mac	***H.similis***
43	Dens with 10 spines	***H.transitoria***
–	Dens with more than 20 spines	**44**
44	Abd. IV centrally with a transverse band	***H.sauteri***
–	Abd. IV centrally without a transverse band	***H.sinensis***
45	Labial basal chaeta E ciliate	***H.leei***
–	Labial basal chaeta e smooth	**46**
46	Th. II posteriorly with a M-shaped transverse stripe	***H.pseudosinensis***
–	Th. II posteriorly without a M-shaped transverse stripe	**47**
47	Labial basal chaeta l_1_ smooth	***H.quadrimaculata***
–	Labial basal chaeta L_1_ ciliate	***H.sichuanensis***

## Supplementary Material

XML Treatment for Homidia acutus

XML Treatment for Homidia changensis

XML Treatment for Homidia linhaiensis

XML Treatment for Homidia socia
